# Widespread modulation of gene expression by copy number variation in skeletal muscle

**DOI:** 10.1038/s41598-018-19782-4

**Published:** 2018-01-23

**Authors:** Ludwig Geistlinger, Vinicius Henrique da Silva, Aline Silva Mello Cesar, Polyana Cristine Tizioto, Levi Waldron, Ralf Zimmer, Luciana Correia de Almeida Regitano, Luiz Lehmann Coutinho

**Affiliations:** 1 Animal Biotechnology Laboratory, Animal Science Department, University of São Paulo (USP)/Luiz de Queiroz College of Agriculture (ESALQ), Piracicaba, CEP 13418-900 Brazil; 2 Embrapa Pecuária Sudeste, São Carlos, CEP 13560-970 Brazil; 3 Institute of Bioinformatics, Department of Informatics, Ludwig-Maximilians-Universität München (LMU), München, 80333 Germany; 4 Graduate School of Public Health and Health Policy, City University of New York, New York, NY 10027 USA; 5 Institute for Implementation Science and Population Health, City University of New York, New York, NY 10027 USA

## Abstract

Copy number variation (CNV) is a frequently observed deviation from the diploid state due to duplication or deletion of genomic regions. Although intensively analyzed for association with diseases and production traits, the specific mechanisms and extent by which such variations affect the phenotype are incompletely understood. We present an integrative study on CNV and genome-wide gene expression in Brazilian Bos indicus cattle. We analyzed CNVs inferred from SNP-chip data for effects on gene expression measured with RNA-seq in skeletal muscle samples of 183 steers. Local effects, where expression changes coincided with CNVs in the respective genes, were restricted to immune genes. Distal effects were attributable to several high-impact CNVs that modulated remote expression in an orchestrated and intertwined fashion. These CNVs were located in the vicinity of major skeletal muscle pathway regulators and associated genes were enriched for proteolysis, autophagy, and muscle structure development. From association analysis between CNVs and several meat quality and production traits, we found CNV-associated expression effects to also manifest at the phenotype level. Based on genome sequences of the population founders, we further demonstrate that CNVs with impact on expression and phenotype are passed on from one generation to another.

## Introduction

Individual genome variation can explain a substantial fraction of variation observed on the phenotypic level. During the past two decades, the analysis of genome variation concentrated especially on single nucleotide polymorphisms^1^ [SNPs]. Up to the present day, millions of SNPs have been detected and tested in numerous genome-wide association studies (GWAS) for effect on various phenotypes such as diseases and production traits^2,3^.

However, it has been observed that genome variation is not limited to SNPs. Larger regions of structural variation such as insertions, inversions and translocations have been reported and are assumed to cover a considerable fraction of the genome^4^. Among the various types of structural variation, copy number variations (CNVs) are frequently observed deviations from the diploid state due to duplication or deletion of genomic regions^5^.

CNVs can be experimentally detected based on comparative genomic hybridization^6^ [CGH]. On the other hand, CNVs can also be computationally inferred from SNP-arrays^7,8^. A frequently used algorithm to infer CNVs from SNP-chip data is PennCNV, which shows good consistency with the CGH gold standard^9,10^. In addition, recent developments have also enabled the detection of CNVs from next-generation sequencing data^11^. However, the extent to which the different methods for CNV detection are replicating or complementing each other is subject of ongoing research^12^.

Intuitively, the number of copies of a gene can have a profound effect on its expression^13^. It has also been shown that expression can be affected by CNVs in the vicinity of genes^14^. However, due to dosage compensation mechanisms, changes in copy number do not always translate into expression changes in a straightforward way^15^. On the other hand, CNV-associated expression changes have been reported to contribute to phenotypic variation such as differences in muscle fiber development^16^.

In this article, we analyze the effect of CNVs on genome-wide gene expression in skeletal muscle of Nelore cattle. We subsequently integrate this information with phenotype data for a range of meat quality and production traits and complement these findings by analyzing whether the detected CNVs can be traced back to the founding sires of the population under study.

## Results

**Figure 1:**
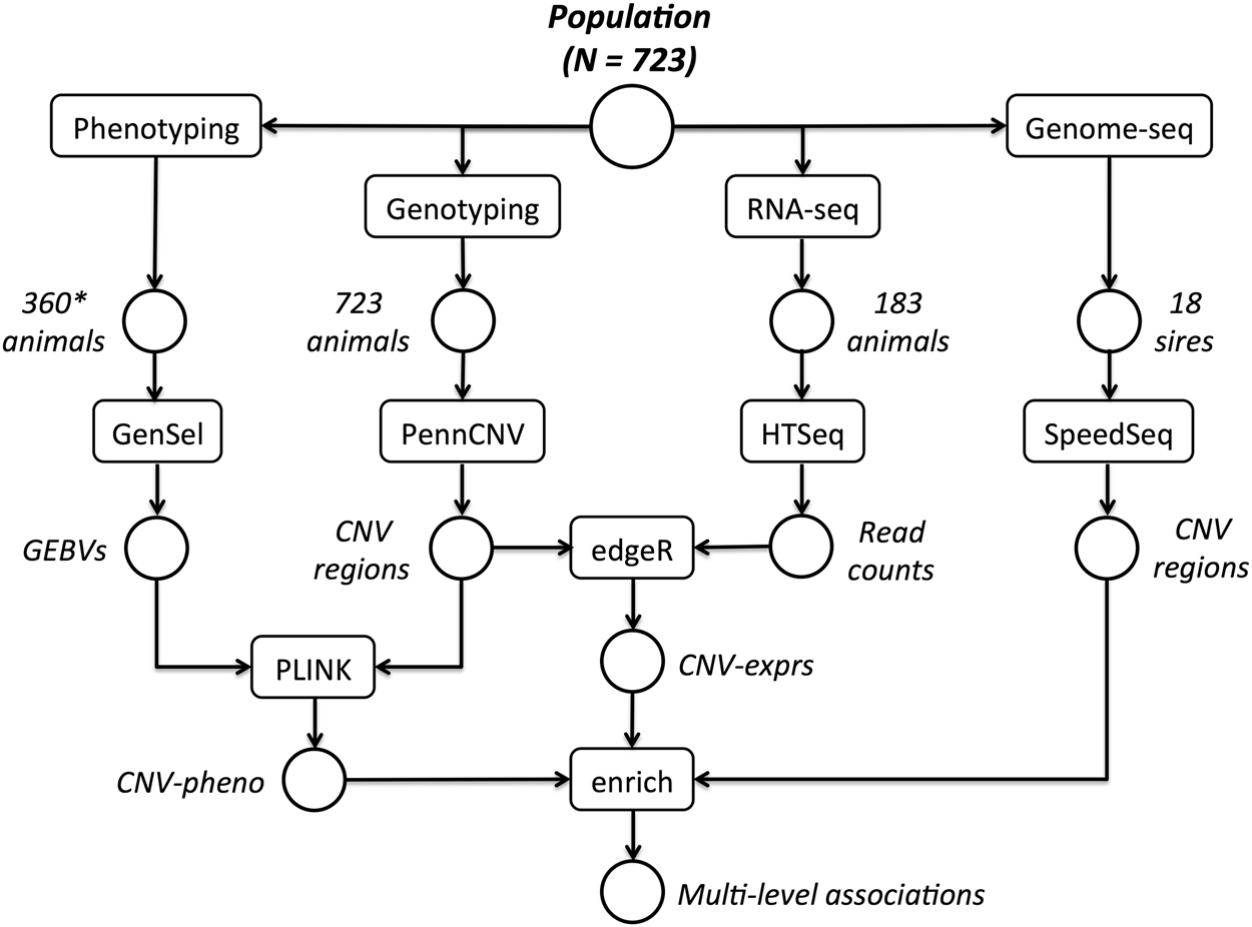
Study setup. The population under study comprises 723 Nelore steers produced by crossing 34 founding sires with commercial dams. All animals were subjected to genotyping and CNV analysis as described previously^17^. A subset of 183 animals were additionally subjected to transcriptome analysis via RNA-seq in samples of skeletal muscle. The RNA-seq read counts were tested for association with the detected CNV regions using edgeR^50^. Resulting CNV-expression associations were enriched with (i) CNV-phenotype associations as obtained with PLINK^61^ from association analysis between CNV regions and genomic estimated breeding values (GEBVs) and (ii) CNV regions as obtained from applying SpeedSeq^63^ to the genomes of 18 of the founding sires. See Material and Methods for details. *Phenotype measurements were obtained for varying fractions of the population as described in Supplementary Table S4.

We have previously reported the genome-wide analysis of copy number variation (CNV) inferred from high-density SNP-chip data for a Nelore population of 723 steers^17^. Here, we integrate the detected CNV regions with (i) genome-wide gene expression data as measured with RNA-seq in muscle samples of 183 steers, (ii) phenotype data on several meat quality and production traits for varying fractions of the population, and (iii) CNV regions detected in the genome sequences of 18 founding sires of the population. For combined analysis of the heterogeneous data sources, we applied an integrative approach that is outlined in Fig. 1.

Assuming distinct modes of action, we divided the effects observed in the CNV-expression analysis into (i) local effects, where expression changes coincide with CNVs in the respective genes and (ii) distal effects, where CNVs supposedly affect trans-acting regulators.

### Local expression effects

After filtering genes for sufficient expression and samples with overlapping CNV calls (see Methods, *Association analysis between CN state and expression*), we tested 61 CNV-containing genes for local effects of CNV on expression (Supplementary Table S1). We observed significant dosage effects for 11 genes including 6 genes of the major histocompatibility complex (MHC), 4 interferon-inducible GTPases (IIGPs), and a paralog of the ATP binding cassette (ABC) transporter *ABCC4*. Of note, these molecular functions have been previously found enriched in genes coinciding with frequently occurring CNVs in diverse cattle breeds^18^. Furthermore, these functions play important roles in protective immunity and adaptation^19–21^.

**Figure 2:**
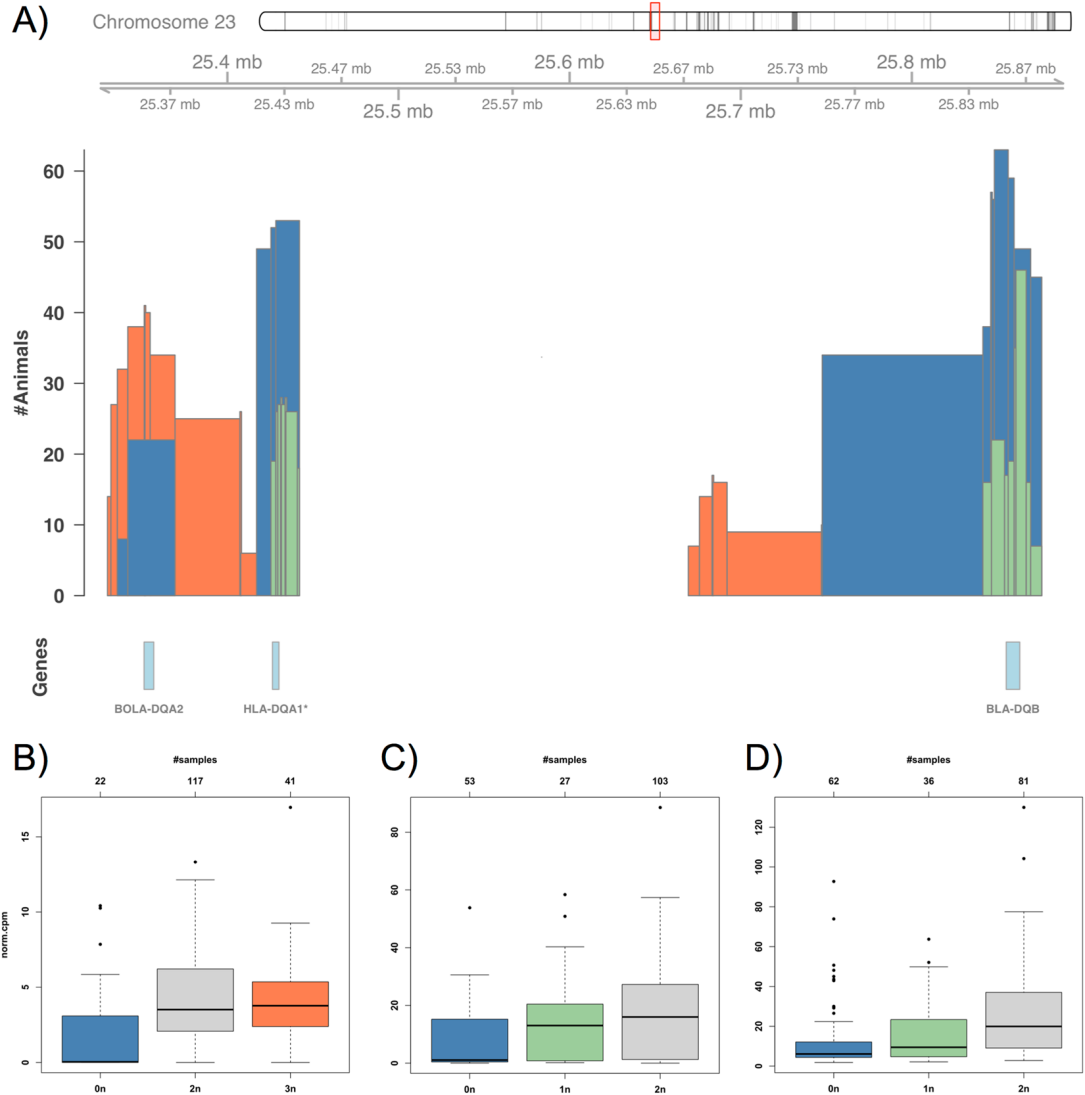
Local effects of copy number variation: significant dosage effects of MHC genes. (**A**) Shown is the region on chromosome 23 harboring the MHC genes BOLA-DQA2, HLA-DQA1* (80% sequence similarity), and BLA-DQB. The histogram depicts the number of animals (*y*-axis) that have been called to contain a complete deletion (0*n*, blue), partial deletion (1*n*, green) or one copy gain (3*n*, orange) in the corresponding regions. The boxplots (**B–D**) show for each gene the expression (*y*-axis, normalized counts per million reads mapped) stratified by CN state (*x*-axis). The number of samples in each CN group is indicated on top of each plot. As an example, BOLA-DQA2 (bottom left in (**A**), *Genes* track) is called as 0*n* in 22 animals and as 3*n* in 41 animals (#*Animals* track in (**A**) and #*samples* axis on top of (**B**)), where the expression of BOLA-DQA2 is found significantly decreased for the blue 0*n* group in (**B**).

Among the significant effects for MHC genes, we observed a particularly striking locus on chromosome 23 harboring ENSBTAG00000037605 (80% sequence similarity with human *HLA*-*DQA1*), *BOLA*-*DQA2*, and *BLA*-*DQB* (Fig. 2a). All three genes show a high degree of variation in copy number for substantial subsets of the population with a significant decrease in expression upon loss of copies (Fig. 2b–d). Similar effects were observed for two additional MHC genes (*JSP*.*1* and *BOLA*, Supplementary Figure S1). A high level of variation within as well as between breeds has been observed before for several MHC genes^19,22^. In previous studies, we have also found *BLA*-*DQB* differentially expressed in genetically divergent sample groups for iron content^23^ and residual feed intake^24^.

Although clearly shifted towards zero expression, we found the genes in Fig. 2b–d expressed in several samples called with encompassing complete deletions (0*n*). While low expression at a completely deleted locus might be attributable to RNA-seq read mismapping, outliers of unexpectedly high expression rather result from inaccurate CNV borders of the SNP-based inference. This is especially apparent for the case of ENSBTAG00000003352 (63% sequence similarity with cattle *HLA*-*DOB*) displaying increased expression for animals called as 0*n* when compared to the 2*n* group (Supplementary Figure S1). However, this indicates intersecting rather than full-spanning deletions, which might not or even positively influence expression of the remaining gene sequence^14,25^.

We further observed counterintuitive negative correlation between copy number and expression for two IIGP genes (Supplementary Figure S2). Such cases have been reported before and explained with varying technical and biological hypotheses such as compensation by buffering or feedback regulation^13,25,26^. Following the approach of Schlattl *et al*.^25^, we also evaluated possible dosage compensation effects associated with gene deletions. Therefore, we specifically examined 9 genes that we found in substantial fractions of the population with full-spanning one copy losses (1*n*). For these loci, we calculated the ratio of median expression in the 1*n* and 2*n* group, and then used bootstrapping to define confidence intervals (Supplementary Figure S4). We found a ratio of 0.5 for *BLA*-*DQB* as assumed for a one copy loss, whereas a 0.75-compensation for *HLA*-DQA1* (see again Fig. 2c and d). We also observed three genes (a 97%-paralog of *RPL10A*, a 74%-ortholog of pig *GBP2*, and *MICALL2*) to be apparently fully compensated.

### Distal expression effects

**Figure 3:**
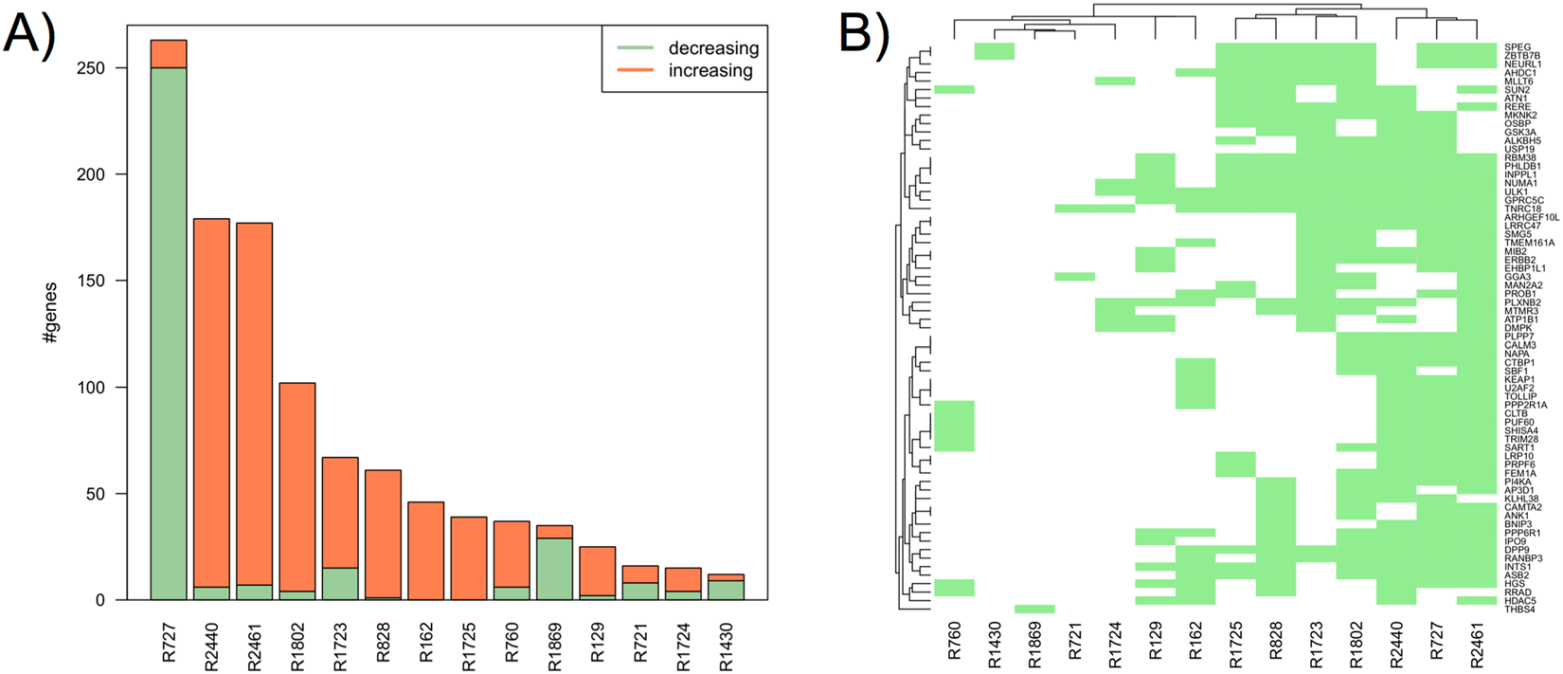
Distal effects of copy number variation: many-to-many relationships associated with consistent expression changes. (**A**) shows the 14 CNV regions (*x*-axis) that were found significantly associated with ≥10 genes (*y*-axis). The colors of the bars indicate how many genes showed either an increase (orange) or decrease (green) in expression with gain in copy number in the corresponding region. As an example, the leftmost bar indicates that expression of almost all R727-associated genes decreased with copy number gain in R727. The clustered adjacency matrix in (**B**) depicts the relationships between the 14 CNV regions (*x*-axis) and the 72 genes that were found significantly associated with ≥3 CNV regions (*y*-axis). For instance, R727 (second region from the right on the *x*-axis) is found associated with almost all of the depicted 72 genes on the *y*-axis. See also Fig. 4 and Supplementary Table S3 for genomic location of the genes and CNV regions.

From testing 173 CNV regions for effects on genome-wide gene expression, we observed 1,087 significant CNV-expression associations between 22 CNV regions and 529 genes (Supplementary Table S2). Among them, we found 14 regions associated each with ≥10 genes. Strikingly, gain in copy number in these regions almost exclusively coincided with either decreased or increased expression of associated genes (i.e. almost all associated genes for a region changed in the same direction, either up or down, as shown in Fig. 3a). As these consistent changes in expression suggested acting of transcriptional regulators, we further investigated these regions. Therefore, we performed a comprehensive functional characterization of each region regarding to (i) co-locating regulatory factors, (ii) transcription factor binding site (TFBS) enrichment at promoters of associated genes, and (iii) functional enrichment of associated genes (Table 1 and Supplementary Table S3).

**Table 1:** Functional characterization of distal CNV regions. Gene names in bold face indicate significant expression changes associated with the corresponding CNV region. Gene names in square brackets indicate location within the corresponding CNV region. Gene names with an asterisk denote gene name annotation by sequence similarity. See Supplementary Table S3 for additional information and Additional File 1, Section 2, for further characterization of each region.

CNV	Coinciding regulators	ChIP enrichment (in CNV region)	TFBS enrichment (of associated genes)	GO enrichment (of associated genes)	Phenotype
R727	[COPS8*], **COPS7A, PHB2, USP5, MLF2, ZNF384**	*not mappable*	**ETS1**, E-Box, **MIZF, HIF1A, USF1**	Proteolysis	Shear force
R2440	**TSC22D4, GNB2, GIGYF1**, COPS6, ZNF394,655,789 MIR25,93,106b	**MAFF, MAFK**	ETS1, **MIZF, CREB1, MAFG**, MAFB, **TP53**	Protein localization	*K* content
R2461	**IPMK**, MIR3924		E-Box, **HIF1A, USF1, MZF1**	Protein transport	Feed efficiency
R1802	**RCN1, LMO2, EIF3M**		**NFIC**, E-Box, USF1	Cell differentiation	Shear force
R828	[LCORL]	**SIX5, SAP30, MAFK**, USF1,2	**MIZF**, FOXO1, SOX10	Anatomic development	Feed efficiency
R162	**ARMC8**		E-Box, **MZF1, USF1**	Muscle development	FA content
R1723-5	PPP1R14C*, JRKL	*depleted*	**NFATC2, AP1**	Protein phosphorylation	Feed efficiency
R760	**[TTC38], ATXN10, FBLN1**	**TCF7L2, TCF12, NRI3C1**, PAX5	**MIZF, TP53**, NFKB	Muscle development `	Shear force
R1869	**RGS2, CDC73**, MIR1278		**PPARG**, SOX10	Myoblast migration	*K* content
R129			TFAP2A, MAFB	Myotube differentiation	Feed efficiency
R721	[7SK RNA], **YBX3**	MEF2C, NFIC, TCF7L2, IRF3, PRDM1, BATF	PAX6	Chromatin organization	Feed efficiency
R1430	EEF1A*	FOS, STAT3	NFIL3, NFYA, AP1	Chromatin organization	Shear force

We first analyzed whether these 14 regions harbor major regulators such as TFs or miRNAs, thereby providing plausible explanations for the observed expression changes. This yielded 4 regions (R721, 727, 760, 828) that contain relevant regulatory factors, which were, however, not sufficiently expressed (i.e. they did not meet the expression threshold applied in the association analysis between CN state and expression). Thus, we subsequently analyzed genes 1 Mb up- and downstream of each region, as CNVs have been found to also affect the expression of nearby genes^14,25^. Except for 2 regions (R129, 828), this revealed CNV-associated expression changes of genes modulating major skeletal muscle pathways such as WNT/*β*-catenin, Akt, and G-Protein signaling^27^. This also underpinned the particularly influential role of R727 and R2440, which we found associated with the most genes in Fig. 3a.

**Figure 4:**
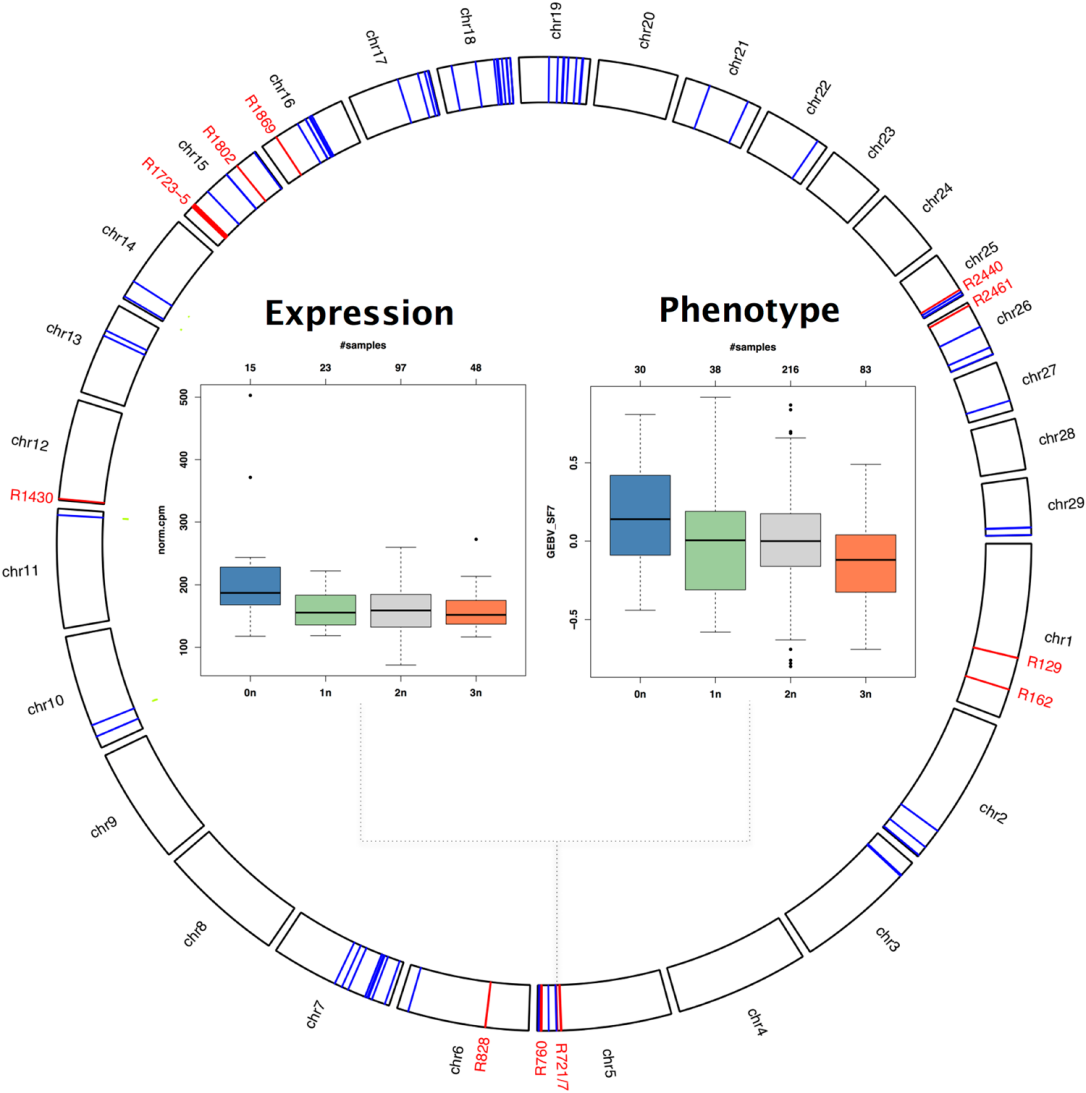
Multi-level associations detected at the interface of copy number variation, gene expression and phenotype. The circle on the outside shows the genomic location of the 14 CNV regions (red) and the 72 most frequently affected genes (blue) from Fig. 3. The boxplot on the left shows the expression of *SCGA* (*y*-axis, normalized counts per million reads mapped) stratified by CN state in R727 (*x*-axis). Sarcoglycan Alpha (*SCGA*) is critical to the stability of muscle fiber membranes and is chosen here as a representative of the various R727-associated genes that display similar expression patterns. The boxplot on the right shows genomic estimated breeding values for shear force (7 days after slaughter, *y*-axis) stratified by CN state in R727 (*x*-axis). The number of samples in each CN group is indicated on top for both boxplots.

Closer inspection demonstrated that a complete deletion (0*n*) in R727 is associated with increased expression of 250 genes (Figs 3a and 4). The region contains a pseudogene of 95% sequence similarity with the *COPS8* subunit of the COP9 signalosome (CSN), which influences protein stability of major TFs^28^. A gene cluster located 0.4–0.7 Mb downstream of R727 contains several additional regulatory factors, including the *COPS7A* subunit of the CSN, for which we found increased expression in R727-0*n* samples (Supplementary Figure S8).

On the other hand, one copy gain (3*n*) in R2440 is associated with increased expression of more than 170 genes. The region resides within a cluster of several microRNAs and zinc finger TFs, but also locates 0.2 Mb upstream of *COPS6*, another subunit of the CSN. Additional regulatory factors in the vicinity of R2440 include the leucine zipper TF *TSC22D4*, G Protein Subunit Beta 2 (*GNB2*), and *GIGYF1*, which acts cooperatively with Grb10 in regulating muscle fiber development^29^. These regulators also showed significantly increased expression in R2440-3*n* samples (Supplementary Figure S16).

As the human genome is considerably better annotated than the cattle genome, we also mapped the detected regions to human and screened them for experimentally validated TFBSs. This revealed several regions, where we found ChIP-seq binding enrichment coinciding with CNV-associated expression changes of specific TFs. Among them is the *LCORL*-containing region R828, which has been repeatedly found associated with body composition and feed efficiency in livestock^30^. TFs enriched at R828 included Six5, a regulator of anatomic development, for which we also found increased expression in the R828-3*n* sample group.

Using a precompiled collection of candidate TFBSs in the cattle genome^31^, we next analyzed enrichment at promoters of associated genes. This revealed for several of the 14 regions an enrichment of E-box motifs, a known binding site for the MyoD master regulator of the skeletal muscle gene expression program^32^. Repeatedly found enriched TFs also included the myogenic differentiation regulator MIZF^33^ and E-box-binding MyoD-competitor Usf1^34^. For R727, we found Ets1 binding sites enriched, which is interesting as changes in CSN activity reportedly affect Ets1 expression and stability^35,36^.

From functional enrichment analysis (see Methods), we found CNV-associated genes involved in core processes of myogenesis and skeletal muscle physiology such as myoblast migration, myotube differentiation, and muscle structure development. This confirmed the specific relevance of each region and was in line with the observed effects on upstream regulators. For instance, we found proteasome proteolytic function enriched among R727-associated genes, which is consistent with studies reporting the CSN and Ets1 as regulators of proteolytic activity in skeletal muscle^37,38^.

Noteworthy, we also observed 72 genes to be associated with ≥3 CNV regions (Fig. 3b). Candidate TFBS analysis of these genes again revealed an enrichment of E-box motifs. We further found these genes functionally enriched for muscle structure development processes and autophagy, which regulates skeletal muscle metabolism^39^. In agreement, we found the serine/threonine kinase *ULK1*, a major regulator of autophagy^40^, associated with 10 CNV regions.

**Figure 5:**
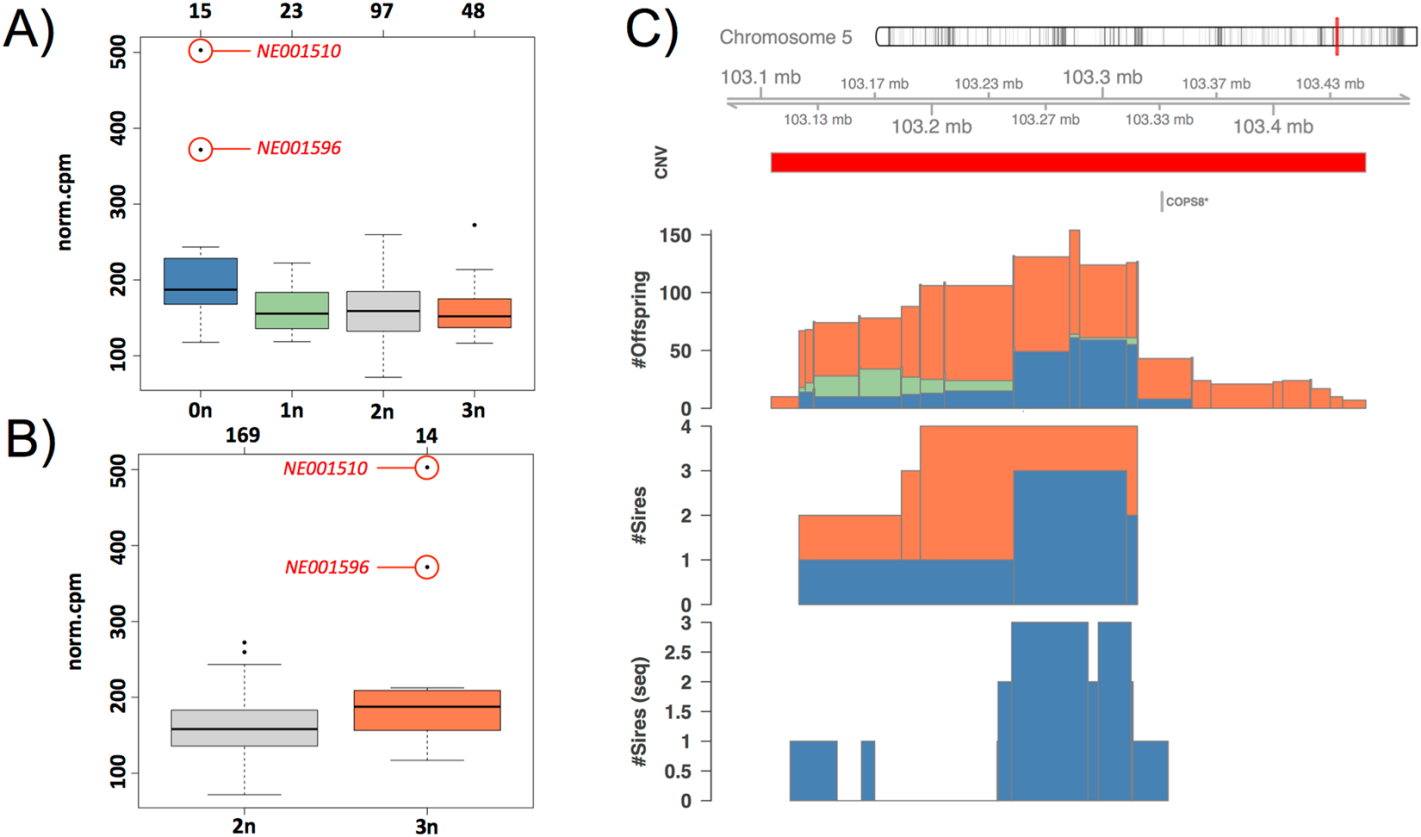
CNV synergy, inheritance and fine-mapping. The boxplots on the left show Sarcoglycan Alpha (*SCGA*) expression as stratified by CN state in (**A**) R727 and (**B**) R2440. Highlighted in red are the two samples carrying both expression-increasing alleles, i.e. 0*n* in R727 and 3*n* in R2440. Shown in (**C**) is the location of R727 (CNV track in red) and the COPS8* pseudogene (ENSBTAG00000047936) on chromosome 5. The #*Offspring* and #*Sires* tracks below show the number of offspring (out of 723) and sires (out of 34), respectively, that have been called by PennCNV to contain a complete deletion (0n, blue), partial deletion (1n, green) or one copy gain (3n, orange) in R727. The #*Sires* (*seq*) track at the bottom shows corresponding deletion calls from the CNV-seq approach. The region 103.28 ± 0.04 Mb that locates immediately upstream of the COPS8* pseudogene shows a peak of 0*n* calls (sample group with increased expression in (**A**)) in the offspring population as well as for the sires when calling CNVs from SNPs and sequencing data.

Further inspection of the many-to-many relationships between CNV regions and associated genes confirmed that the regions associated with the most genes such as R727 and R2440 (Fig. 3a) share a large number of associated genes (Fig. 3b). Whereas samples carrying either 0*n* for R727 or 3*n* for R2440 individually displayed increased expression of associated genes, we found a strong synergistic effect for samples carrying both expression-increasing alleles (Fig. 5a and b).

### Phenotype effects

Having detected local and distal CNV-expression associations, we next investigated whether these effects can also be detected on the phenotype level. Therefore, we analyzed phenotype data on several meat quality and production traits such as meat tenderness, feed efficiency as well as mineral and fatty acid content (full list of analyzed phenotypes is described in Supplementary Table S4). Interestingly, we found that the expression effects observed for several CNV regions closely resembled the effects observed on the phenotype level (Supplementary Figures S18–S24).

This includes R727, for which we have previously found a significant association with meat tenderness^17^. As illustrated in Fig. 4, a complete deletion (0*n*) in R727 is not only associated with increased expression, but also with increased genomic estimated breeding values (GEBVs) for shear force measured 7 days after slaughter. With regard to tenderness, we observed 3 additional regions associated with shear force and 2 regions with potassium content (reported marker of tenderness^41^). On the other hand, we found 7 regions associated with feed efficiency as measured by the feed conversion ratio of dry matter intake and average daily gain. Several regions were also associated with fatty acid content (Supplementary Table S3). We accordingly found genes with CNV-associated expression changes enriched for genes that we have previously found differentially expressed in genetically divergent sample groups for oleic acid content^42^.

As the phenotyped population for the analyzed traits was up to twice as large (≈360 animals) as the transcriptomically analyzed population (≈180 animals), we observed that expression effects became more pronounced on the phenotype level. For instance, samples called as 3*n* in R727 displayed for most associated genes no or only slightly decreased median expression, whereas median shear force GEBV was significantly decreased (see again Fig. 4). In addition, several regions with bi-allelic expression changes displayed extended multi-allelic effects on the phenotype level (Supplementary Figures S21–S23). For instance, R1723 shows a bi-allelic (2*n*/3*n*) expression change and an extended tri-allelic (1*n*/2*n*/3*n*) phenotype effect in Supplementary Figure S21.

### Inheritance and fine-mapping

Having detected CNVs associated with expression changes and phenotype variation, we next investigated whether these effects could be traced back to the population founders. Therefore, we separately fine-mapped CNV calls for offspring and sires within CNV regions. This revealed that (i) offspring calls peaked around the presumed actual breakpoints, and (ii) that the observed patterns were typically recovered for the sires (Supplementary Figures S5–S17). For instance, fine-mapping of R727 in offspring and sires concordantly highlighted a region at 103.28 ± 0.04 Mb that locates immediately upstream of the COPS8* pseudogene (Fig. 5c).

In addition to the SNP-based calls, we also analyzed CNV calls derived from sequencing of the sire genomes. Median concordance of SNP-based calls with sequencing-based calls was 77.5% across sires (Supplementary Figure S25a). This decreased to 68.1% when also requiring CNV type (deletion/duplication) to be consistent. However, taking known problematic aspects of identifying duplications with sequencing-based approaches into account^43^, and thus restricting evaluation to deletions, resulted in a median concordance of 85.6% (Supplementary Figure S25b). Accordingly, case-specific inspection of sequencing-based calls in the 14 distal CNV regions typically confirmed CNV borders, whereas CNV type agreed for deletions but disagreed for several cases of SNP-inferred duplications (Supplementary Figures S5–S17). On the other hand, revisiting local effects, where we observed gene deletions (0*n*) with apparent expression as for ENSBTAG00000003352, allowed to better resolve CNV borders with sequencing-based calls, which supported the hypothesis of intersecting, rather than encompassing deletions (Supplementary Figure S3).

## Discussion

CNV is a major type of structural genome variation, for which an increasing number of studies also indicate association with gene expression and phenotypic traits. To investigate how and to which extent CNV contributes to expression and phenotype diversity within a population, we addressed the following questions:

- By which mechanisms are CNVs influencing gene expression?
- Is CNV-induced expression variation translated to the phenotype level?
- Do CNVs with influence on expression and phenotype arise *de novo* or can they be traced back to the population founders?

Therefore, we analyzed a large population of Brazilian *Bos indicus* cattle, which has been systematically genotyped, subjected to transcriptome quantification in skeletal muscle, and phenotyped with respect to various meat quality and production traits.

For the first question, we assumed CNV-expression associations to either result from (i) local CNVs coinciding with genes or (ii) distal CNVs that influence gene expression from remote sites of the genome.

We observed only a limited number of genes for which coinciding CNVs resulted in detectable differences in expression. This extends previous findings that have found genes depleted for CNVs^4,17^. However, several MHC, IIGP and ABC transporter genes displayed significant dosage effects. As these functions are important for protective immunity and adaptation, a high level of variation is essential for generating host immune responses^19–21^. Previous CNV studies in cattle have also found population- and breed-specific differences for CNVs in these genes^44,45^. As indicated by our findings, these differences are presumably also present on the expression level.

In contrast to the limited number of local CNV effects, we have observed a variety of long-range CNV-expression associations. The majority of these associations were attributable to several high-impact CNV regions, each associated with orchestrated expression changes of ≥10 genes. When further investigating these regions, we found them to locate in the vicinity of important regulators of major skeletal muscle pathways. Such neighborhood effects of CNVs have previously been explained by disruption or modification of the local chromatin structure^14,25^. When mapping these regions to human and analyzing experimentally validated TF binding, we found specific TFs enriched and with CNV-associated expression changes. These findings are consistent with a model where CNVs disrupt or modify regulatory elements. Assuming a substantial fraction of species-specific regulatory elements, additional insights are expected to come from improved functional annotation of the cattle genome by the FAANG project^46^.

From candidate TFBS and functional enrichment analysis of associated genes, we further characterized downstream affected regulators and processes. The detected enrichment of E-box motifs suggested acting of the MyoD master regulator. This was supported by enriched modulators of MyoD-dependent expression such as MIZF and Usf1. On the other hand, we found CNV-associated genes enriched for core processes of myogenesis and skeletal muscle physiology confirming relevance of the observed expression changes of upstream regulators. Interestingly, we also found a considerable number of genes associated with ≥3 CNV regions. For instance, autophagy-regulating *ULK1* was associated with 10 CNV regions. This indicates complex regulation of *ULK1*, in which CNVs modulate the interplay of several regulatory factors.

CNV interplay was also apparent in the synergy of R727 and R2440, the two regions with the largest, and partly shared, sets of associated genes. Synergistic effects typically indicate simultaneous perturbation of interacting proteins in a protein complex or regulatory pathway^15^. We found a chain of evidence that synergy of R727 and R2440 likely involves the COP9 signalosome (CSN) as (i) both regions locate proximal to CSN subunits, which showed concordant expression increase, (ii) associated genes were enriched for known binding sites of Ets1, reportedly affected by changes in CSN activity, and (iii) associated genes were enriched for proteolytic function, which is regulated by the CSN and Ets1. We also found CNV calls in R727 concentrated immediately upstream of a pseudogene with high similarity to another CSN subunit. This prompts targeted follow-up studies to clarify whether pseudogene-based regulation^47^ also contribute to the observed R727-associated expression changes.

From analysis of meat quality and production traits, we found expression effects observed for high-impact CNV regions closely resembled on the phenotype level. Affected traits included shear force, feed efficiency as well as mineral and fatty acid content. Variation in shear force indicates differences in muscle tissue composition, which we found associated with changes in expression and, thus, presumably also activity of proteolysis and autophagy. Increased expression of structural proteins such as Sarcoglycan Alpha, synergistically affected by CN state in R727 and R2440, also indicated enhanced muscle fiber stability. On the other hand, variation in feed efficiency indicates differences in how feed is converted to skeletal muscle mass. Our findings suggest that involved CNVs affect essential steps of myogenesis such as myotube differentiation, but also loci driving anatomic development. Among them was the *LCORL*-containing region R828, which has repeatedly been found associated with body composition and feed efficiency in livestock^30^. Previous studies on feed efficiency have identified several SNPs in *LCORL*, but found it difficult to establish a clear-cut relation to transcript abundance of *LCORL* (see^30^ for a review and Supplementary Figure S11b, indicating no expression change in R828-3*n* samples). Our findings rather suggest that the detected SNPs are part of a CNV influencing binding of anatomic development regulator Six5. This was supported by enriched binding of Six5 in R828 and enrichment of anatomic development in R828-associated genes. As we also observed increased expression of Six5 in R828-3*n* animals, presumably caused by R828-independent mechanisms, there seems to be a simultaneous increase of Six5 binding sites in R828 and general Six5 availability in these samples. However, further targeted investigation is needed to clarify the mechanisms and whether the region indeed acts as an enhancer on a downstream effector of Six5.

From combined analysis of SNP- and sequencing-based CNV calls, we found that CNVs could often be traced back to the founding sires. As CNV frequency in the offspring population already suggested, analysis of the sires largely recovered CNVs and also confirmed specific patterns within CNV regions. Incorporation of sequencing-based calls thereby facilitated validation and improved resolution of CNV borders. However, it should be noted that straightforward interpretation was impaired by technical and methodological aspects, such as imprecise borders of SNP-inferred CNVs or difficulty in detecting duplications with sequencing approaches. Thus, even when applying well-established tools for CNV analysis, cautious interpretation involving CNV fine-mapping and application of complementary approaches is necessary.

We conclude that our results consolidate CNVs as important modulators of gene expression. Whereas direct effects on gene dosage were restricted to several immune genes, where high variability is essential to maintaining effectiveness of the immune response, we found a variety of long-range CNV effects that modulated gene expression in an orchestrated and intertwined fashion. Closer inspection revealed that these CNVs apparently bear the potential to fine-tune core regulators located in their vicinity, propagating these effects to genome-wide gene expression. This illustrates an important aspect of how genome composition is translated to phenotype variation, as we found CNV-associated expression to manifest at the phenotype level. Of considerable importance for breeding programs, our results imply that CNVs influencing expression and also phenotypic traits such as meat quality and feed efficiency are passed on from one generation to another. However, this is presumably also of importance in the context of disease phenotypes, as we expect CNVs to likewise contribute to pathologic abnormalities in skeletal muscle.

## Methods

### Ethical statement

All experimental procedures involving steers in this study were performed in accordance with the relevant guidelines (Protocol CEUA 01/2013) as approved by the Institutional Animal Care and Use Committee (IACUC) from the Brazilian Agricultural Research Corporation (EMBRAPA) and sanctioned by the president Dr. Rui Machado.

### Population, genotyping and CNV analysis

The population under study comprises 723 Nelore steers, produced by crossing 34 founding sires with commercial dams. All animals were subjected to genotyping and CNV analysis as described previously^17^.

### RNA-seq

Paired-end sequencing using the Illumina HiSeq2500 platform was performed as described previously^42^. Reads were mapped via Tophat2/Bowtie2^48^ using the masked UMD3.1 genome assembly as reference. HTSeq-count^49^ was applied for obtaining read counts.

### Association analysis between CN state and expression

Association testing between the detected CNV regions and RNA-seq read counts was carried out using edgeR, which applies generalized linear models (GLMs) based on the negative-binomial distribution while incorporating normalization factors for different library sizes^50^. In the case of only one CN state deviating from 2*n* for a CNV region under investigation, this reduced to the classical 2-group comparison. For more than two states (e.g. 0*n*, 1*n*, 2*n*), edgeR’s ANOVA-like test was applied to test all deviating groups for significant expression differences relative to 2*n*. To avoid artificial effects due to low expression of a gene or insufficient sample size in deviating groups, we excluded from the analysis (i) genes with fewer than *r* reads per million reads mapped (cpm, counts per million) in the maximally expressed sample group, and (ii) CNV regions with fewer than *s* samples in a group deviating from 2*n*. As local effects had a clear biological indication and number of genes tested were small, we chose thresholds *r* = 3, *s* = 4, and a nominal significance level of 0.05. Due to power considerations and to avoid detection of spurious effects, we chose thresholds *r* = 25 and *s* = 10 for distal effects and carried out multiple testing correction following the procedure in^14^ using an adjusted significance level of 0.01.

### Enrichment analysis

Enrichment analysis of ChIP-seq TF-binding was carried out for each CNV region by (i) mapping to the corresponding region in the human hg19 genome assembly using liftOver^51^, (ii) counting the number of ChIP-seq experiments supporting binding of a TF according to ENCODE’s factorbook^52^, and (iii) assessing statistical significance of the observed evidence as compared to 1000 randomly sampled genomic regions of the same size and chromosome using the regioneR package^53^. CNV-associated genes were tested for TFBS enrichment using a precompiled collection of candidate TFBSs in the cattle genome^31^. Fisher’s exact test was applied to assess statistical significance of the observed number of genes predicted to be bound by a specific TF. GO-BP enrichment of CNV-associated genes was tested using DAVID^54^. Multiple testing correction for the enrichment analysis of ChIP-seq TFBSs, candidate TFBSs, and GO-BP terms was carried out using the method of Benjamini and Hochberg^55^ with an FDR cutoff of 0.1.

### Association analysis between CN state and phenotype

Phenotype measurements were obtained for varying fractions of the population (ranging from 285 to 373 animals, Supplementary Table S4) and transformed to genomic estimated breeding values (GEBVs) using the BayesB method from GenSel^56^ as described previously^57–60^. Association analysis between CNV regions and GEBVs were carried out using PLINK^61^ as described previously^17^. Multiple testing correction was carried out using the method of Benjamini and Hochberg^55^ with an FDR cutoff of 0.1.

### Genome sequencing and CNV analysis

Genome sequencing was carried out for 18 out of 34 founding sires of the population under study. They were chosen based on the number of phenotyped and transcriptomically analyzed offspring in the population. Paired-end sequencing using Illumina HiSeq. 2500 was performed as described for RNA-seq. Reads smaller than 65 bp were removed. Remaining reads were aligned against the UMD3.1 assembly using BWA-MEM with default options^62^. Summary statistics on coverage obtained and reads mapped per sire can be found in Supplementary Table S5. CNV calling was carried out based on SpeedSeq^63^, which incorporates LUMPY^64^ and CNVnator^65^ for CNV detection. Calls with length <1 kb or 1 Mb were excluded. In accordance with the application guidelines, a *q*0-filter of 0.5 for CNVnator and a minimum quality threshold of 10 for LUMPY was applied. Regions of the reference genome with artificially high sequencing depth over multiple individuals were excluded as described previously^64^.

### Data availability

The SNP-based CNV calls analysed in this study are publicly available from the supplementary material in^17^. The RNA-seq dataset is publicly available in the European Nucleotide Archive (ENA, EMBL-EBI) under accession PRJEB13188. All additional datasets generated and analysed during this study are available from the corresponding author on reasonable request.

## Electronic supplementary material

Additional File 1

Additional File 2
