# Supplementary material for: Widespread modulation of gene expression by copy number variation in skeletal muscle
Source: Sci Rep. 2018 Jan 23;8:1399. doi: 10.1038/s41598-018-19782-4 (PMC5780461; doi:10.1038/s41598-018-19782-4)
Supplement: Supplementary file 1 — Additional File 1 [file 41598_2018_19782_MOESM1_ESM.pdf]

Widespread modulation of gene expression by copy number variation in skeletal muscle

Ludwig Geistlinger, Vinicius Henrique da Silva, Aline Silva Mello Cesar,
Polyana Cristine Tizioto, Levi Waldron, Ralf Zimmer,
Luciana Correia de Almeida Regitano, and Luiz Lehmann Coutinho

Supplementary Material

Contents

1 Local CNV effects **2**

2 Distal eCNV regions **6**
2.1 R129 6
2.2 R162 7
2.3 R721 8
2.4 R727 9
2.5 R760 10
2.6 R828 11
2.7 R1430 11
2.8 R1723-5 11
2.9 R1802 11
2.10 R1869 13
2.11 R2440 13
2.12 R2461 14

3 Phenotype effects **16**

List of Figures

S1 Gene dosage effects 1 2
S2 Gene dosage tendencies 2 3
S3 Fine-mapping of ENSBTAG00000003352 4
S4 Dosage compensatory effects 5
S5 Fine-mapping of R129 6
S6 Fine-mapping of R162 7
S7 Fine-mapping of R721 8
S8 R727-associated regulatory factors 1 9
S9 R727-associated regulatory factors 2 10
S10 Fine-mapping of R760 12
S11 Fine-mapping of R828 13
S12 Fine-mapping of R1430 14
S13 Fine-mapping of R1725 15
S14 Fine-mapping of R1802 16
S15 Fine-mapping of R1869 17
S16 Fine-mapping of R2440 18
S17 Fine-mapping of R2461 19
S18 Multi-level associations 1 20
S19 Multi-level associations 2 21
S20 Multi-level associations 3 22
S21 Multi-level associations 4 23
S22 Multi-level associations 5 24
S23 Multi-level associations 6 25
S24 Multi-level associations 7 26
S25 Concordance of snp- and seq-based CNV calls 27

1 Local CNV effects

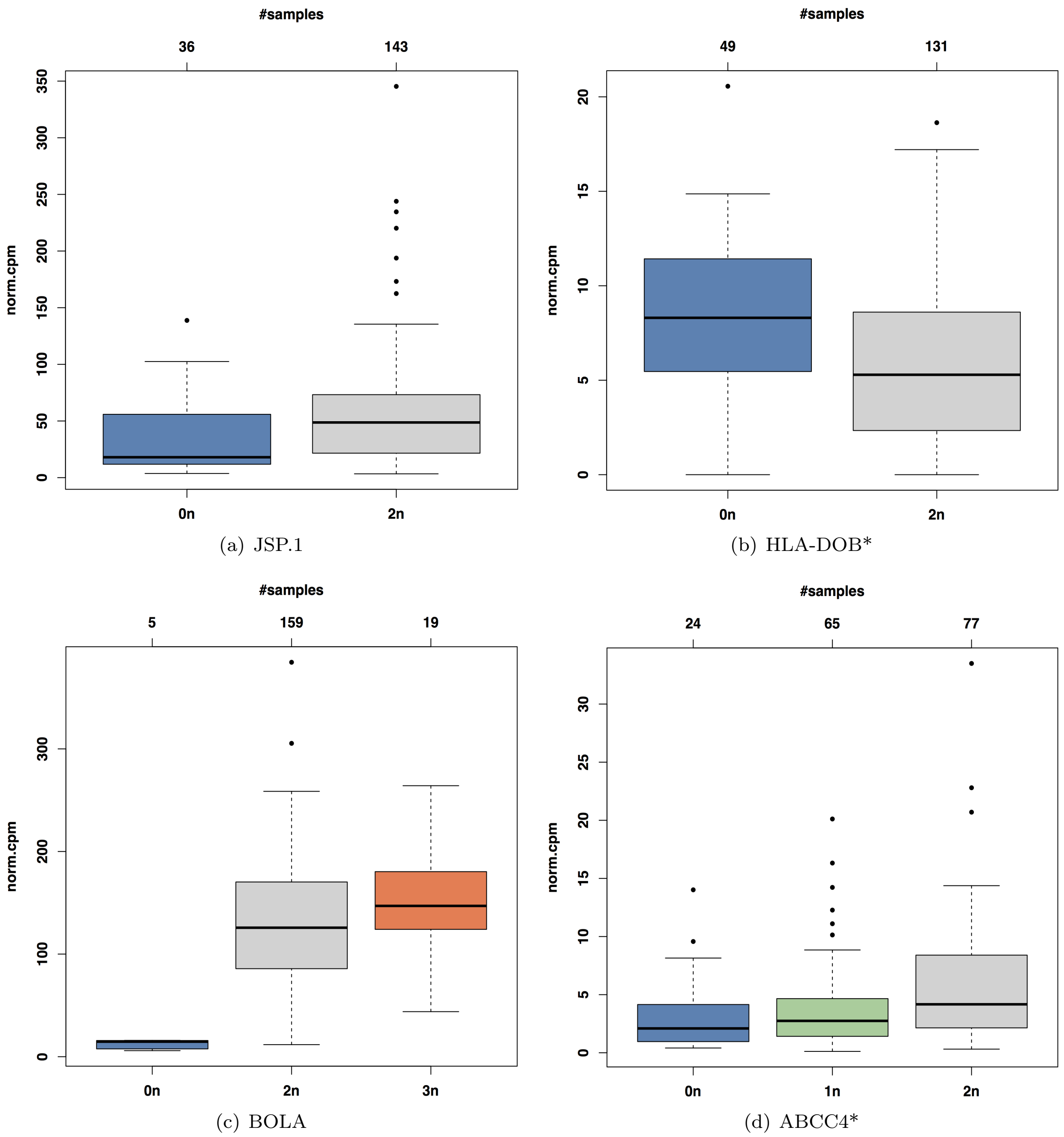

(a) JSP.1 (b) HLA-DOB* (c) BOLA (d) ABCC4*

Figure S1: Significant gene dosage effects. *HLA-DOB**: ENSBTAG00000003352, 63% sequence similarity with cattle *HLA-DOB*. *ABCC4**: ENSBTAG00000045751, 73% sequence similarity with cattle *ABCC4*. See Supplementary Table S1 for further information.

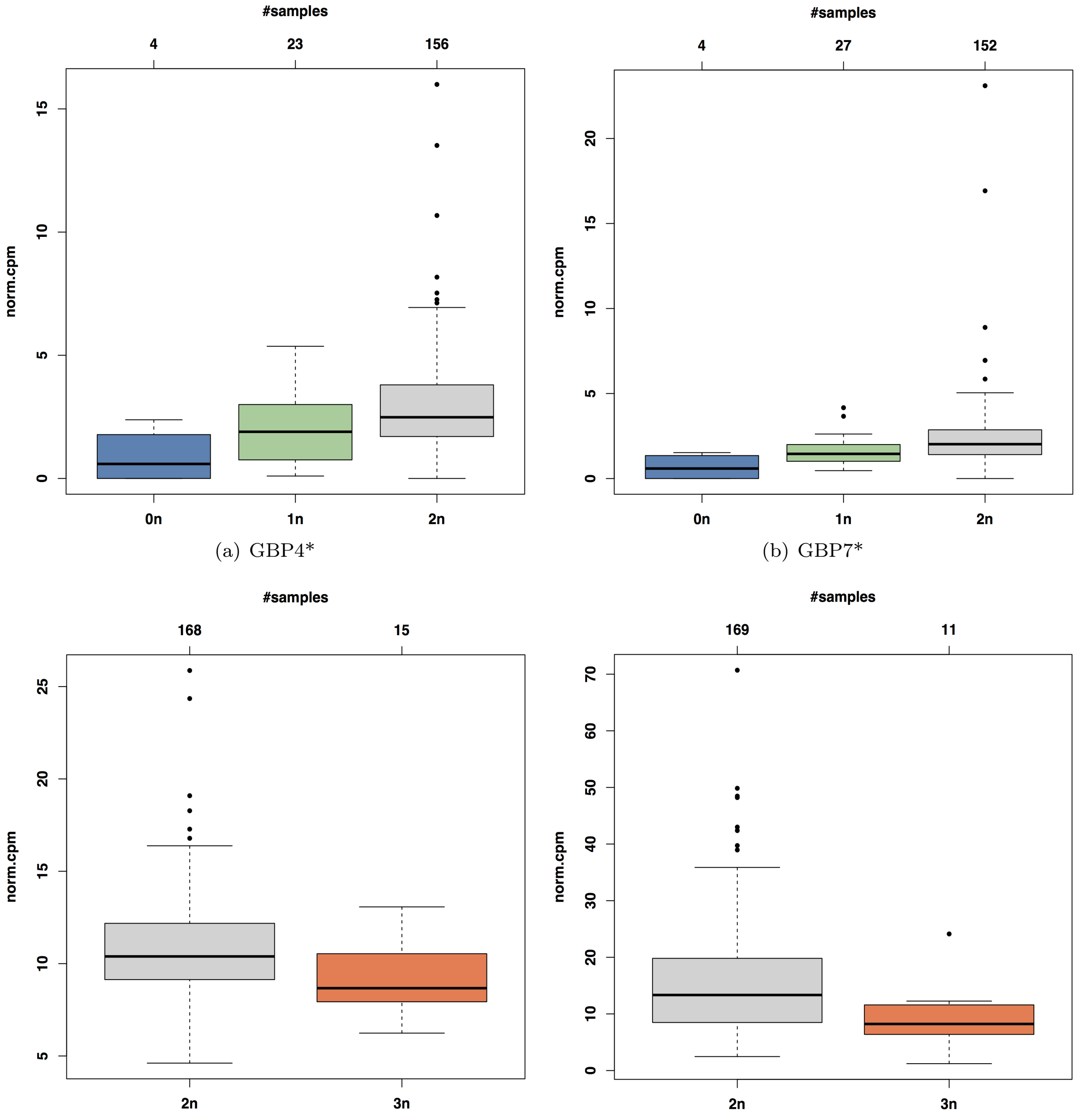

(a) GBP4*

(b) GBP7*

(c) ARL3

(d) GVIN1*

Figure S2: Significant gene dosage effects. *GBP4**: ENSBTAG00000038625, 70% sequence similarity with human *GBP4*. *GBP7**: ENSBTAG00000002416, 70% sequence similarity with macaque *GBP7*. *GVIN1**: ENSBTAG00000037937, 70% sequence similarity with mouse *GVIN1*. See Supplementary Table S1 for further information.

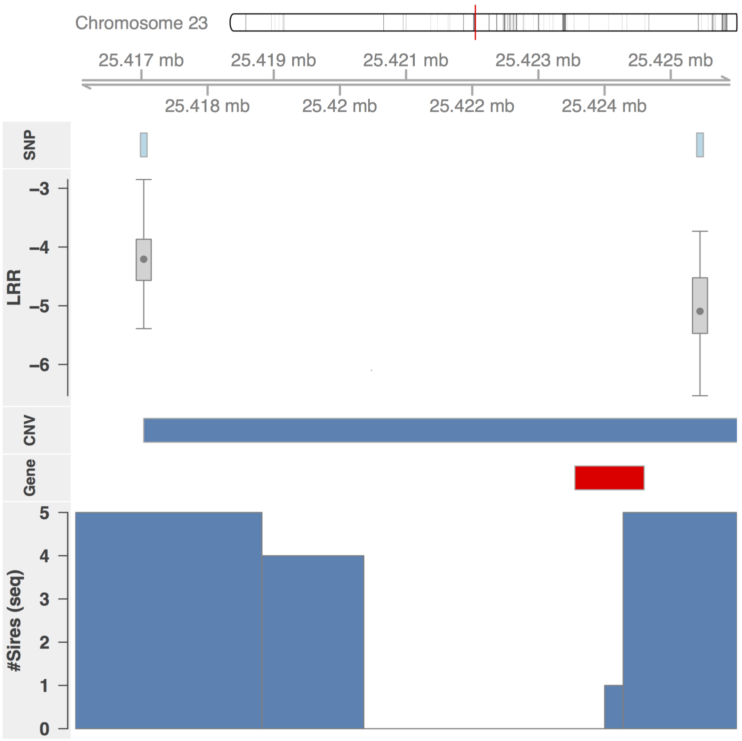

Figure S3: Fine-mapping of CNV calls on ENSBTAG00000003352. The gene track in red shows the location of ENSBTAG00000003352 (63% sequence similarity with cattle *HLA-DOB*). The gene was found to be expressed in animals called with encompassing complete deletions (Figure S1b). The tracks above show the location of the closest SNPs up- and downstream, the corresponding LRR values for all 49 animals called as $0n$, and the resulting `PennCNV` call. This demonstrates that no SNP contributing to the call locates directly on the gene. The track below shows that sequencing-based calls in up to 5 sires support the hypothesis of an intersecting rather than full-spanning deletion.

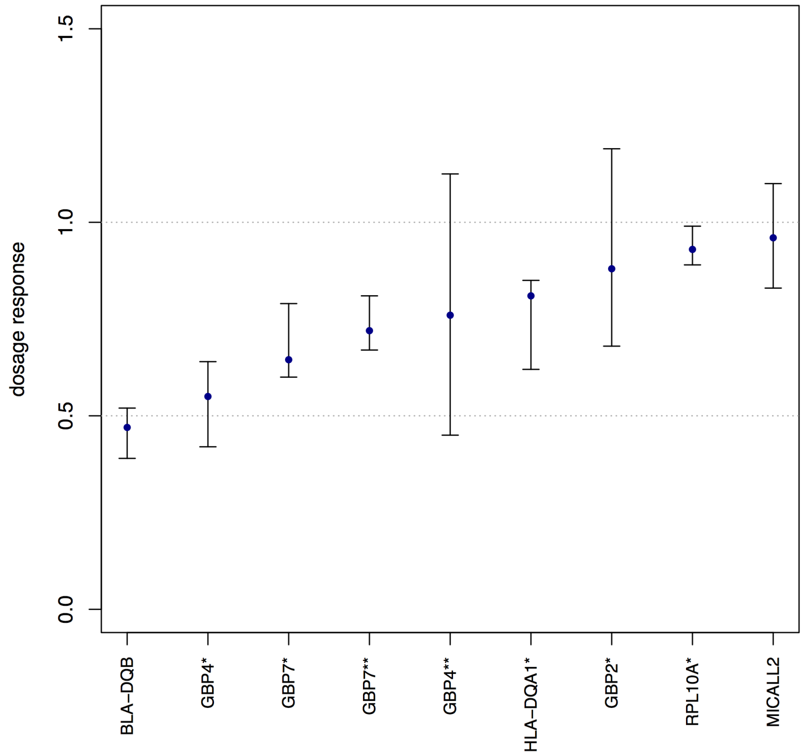

Figure S4: Evaluation of dosage compensation at partial gene deletion loci (1n). The circles mark the ratio between the median expression of samples with CN = 1 and samples with CN = 2. The error bars indicate bootstrapped 95% confidence intervals. *GBP2**: ENSBTAG00000037634, 74% sequence similarity with pig *GBP2*. *GBP4**: ENSBTAG00000024272, 68% sequence similarity with human *GBP4*. *GBP4***: ENSBTAG00000038625, 70% sequence similarity with human *GBP4*. *GBP7**: ENSBTAG00000002416, 70% sequence similarity with macaque *GBP7*. *GBP7***: ENSBTAG00000014529, 67% sequence similarity with mouse *GBP7*. *HLA-DQA1**: ENSBTAG00000037605, 80% sequence similarity with human *HLA-DQA1*. *RPL10A**: ENSBTAG00000027213, 97% sequence similarity with cattle *RPL10A*.

2 Distal eCNV regions

2.1 R129

This is a bi-allelic region on chromosome 1. Fine-mapping highlights a region at 102.9 ± 0.02 Mb. As depicted at the bottom of Figure S5, `CNVnator` calls indicate that this is rather a copy number loss, which is consistent with the findings of Hou *et al.*, 2011 [1].

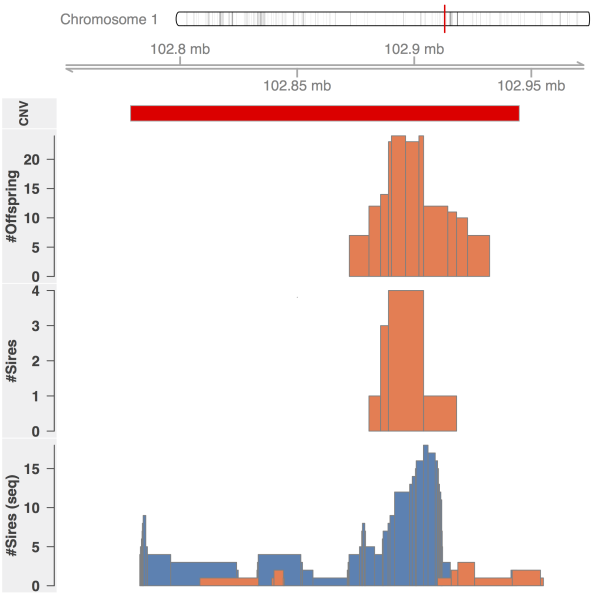

Figure S5: Shown is the location of R129 (CNV track in red) on chromosome 1. The two data tracks below show the number of offspring and sires, respectively, that have been called by `PennCNV` to contain a one copy gain (3n, orange) in R129. The third track at the bottom shows corresponding CNV-seq calls from `CNVnator` (deletions in blue and duplications in orange).

2.2 R162

This is a bi-allelic region on chromosome 1. Fine-mapping highlights a region at 132.03 $\pm$ 0.01 Mb (Figure S6a). Although we found no corresponding `PennCNV` calls in the founding sires, CNV-seq calls from `CNVnator` in up to 5 of the sires confirmed location and CNV type (gain) of this region. The region locates immediately upstream of the Armadillo Repeat Containing 8 (*ARMC8*, reverse strand) gene, for which we found slightly decreased expression in the $3n$ group (Figure S6b).
Proteasomal degradation of α-catenin is regulated by interaction with *ARMC8* [2]. Degradation of α-catenin regulates β-catenin signaling [3], where WNT/β-catenin signaling regulates myogenesis in a variety of ways [4].

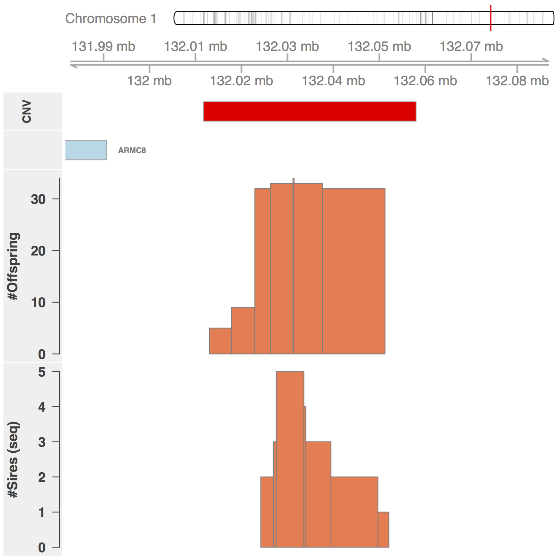

(a) Fine-mapping of R162

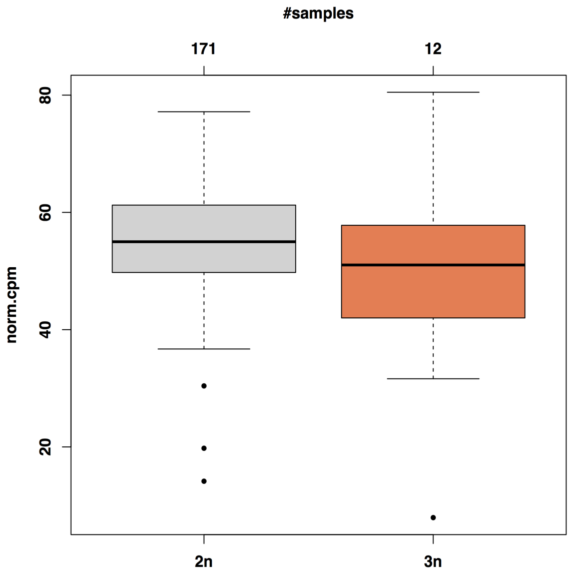

(b) *ARMC8* expression

Figure S6: (a) shows the location of R162 (CNV track in red) and the *ARMC8* gene on chromosome 1. The data track below show the number of offspring that have been called by `PennCNV` to contain a one copy gain (3n, orange) in R162. The data track at the bottom shows corresponding CNV-seq calls from `CNVnator`. The boxplot in **(b)** shows the expression of *ARMC8* (y-axis, normalized counts per million reads mapped) stratified by CN state (x-axis). The number of samples in each CN group is indicated on top of the plot.

2.3 R721

This is a multi-allelic region on chromosome 5. Fine-mapping highlights a region at 99.56 $\pm$ 0.02 Mb, which contains 7SK RNA (Figure S7a). 7SK RNA has been shown to negatively regulate transcription factor *P-TEFβ* [5], which is an activator of *MyoD*-dependent transcription [6].
The region also locates 0.16 Mb downstream of *YBX3*, a transcription factor that represses myogenin transcription in skeletal muscle [7]. We found changes in expression of *YBX3* to associate with changes in CN state of R721 (Figure S7b).

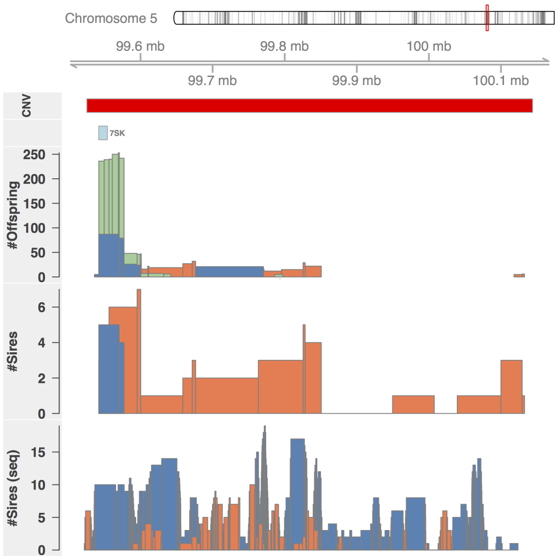

(a) Fine-mapping of R721

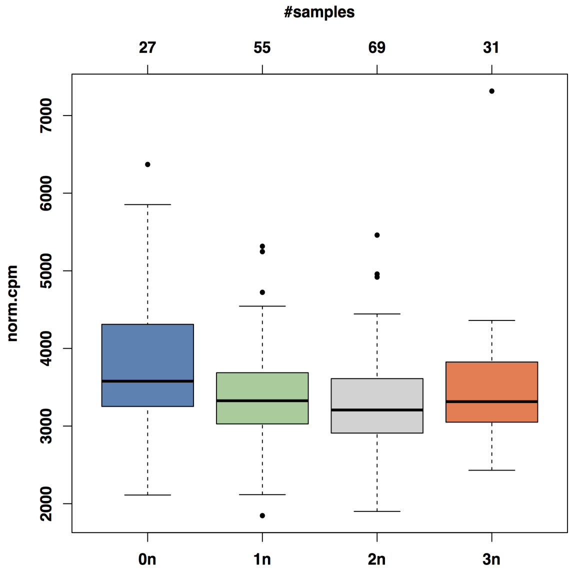

(b) *YBX3* expression

Figure S7: (a) shows the location of R721 (CNV track in red) and the 7SK RNA on chromosome 5. The data tracks below show the number of offspring and sires, respectively, that have been called by `PennCNV` to contain a one copy gain (3n, orange) in R162. The data track at the bottom shows corresponding CNV-seq calls from `CNVnator`. The boxplot in **(b)** shows the expression of *YBX3* (y-axis, normalized counts per million reads mapped) stratified by CN state (x-axis). The number of samples in each CN group is indicated on top of the plot.

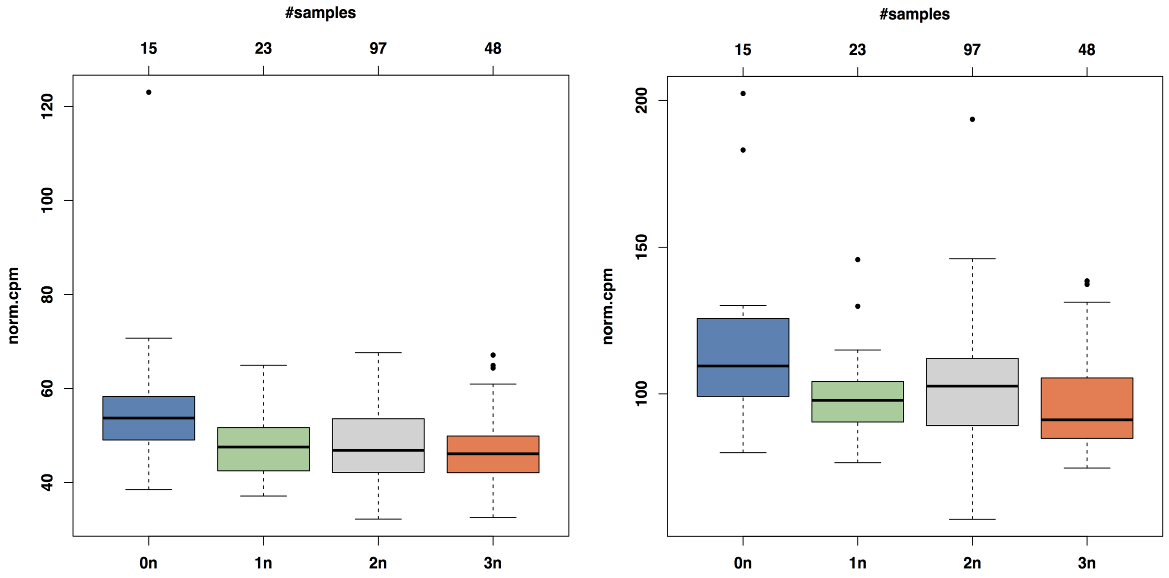

(a) COPS7A (b) PHB2

Figure S8: Regulatory factors associated with R727.

2.4 R727

This is a multi-allelic region on chromosome 5. Fine-mapping highlights a region at 103.28 $\pm$ 0.05 Mb, which is immediately upstream of subunit 8 of the COP9 signalosome complex (*COPS8*, see Figure 5a of the main paper). Whereas the implications of the COP9 signalosome complex are explained in the main paper (see Results section *Distal effects*), we found the expression of several additional regulatory factors that locate in the vicinity of R727 to be also associated with CN state in R727. This includes another subunit of the COP9 complex, *COPS7A*, as well as *PHB2*, *USP5*, *ING4*, *MLF2*, and *ZNF384* (Figure S8 and S9). These genes locate in a gene cluster 0.4-0.7 Mb downstream of R727.

Prohibitin 2, *PHB2*, interacts with both *MyoD* and *MEF2*, and represses both *MyoD*- and *MEF2*-dependent gene transcription [8].

Knock-down of Ubiquitin Specific Peptidase 5, *USP5*, causes the accumulation of *TP53* and, thus, an increase in *TP53* transcriptional activity. *TP53* is a master regulator, which has also been shown to regulate muscle differentiation at the myogenin step [9].

Inhibitor Of Growth Family Member 4, *ING4*, has transcription co-activator activity and binds *TP53* and *EP300*, an important co-factor of *MyoD*-dependent transcription [10].

Myeloid Leukemia Factor 2, *MLF2*, suppresses p27Kip1, a master regulator of skeletal muscle growth [11].

Zinc Finger Protein 384, *ZNF384*, is a transcription factor that binds and regulates the promoters of several matrix metallopeptidases, which have important physiological functions in maintenance of the integrity and homeostasis of muscle fibers [12].

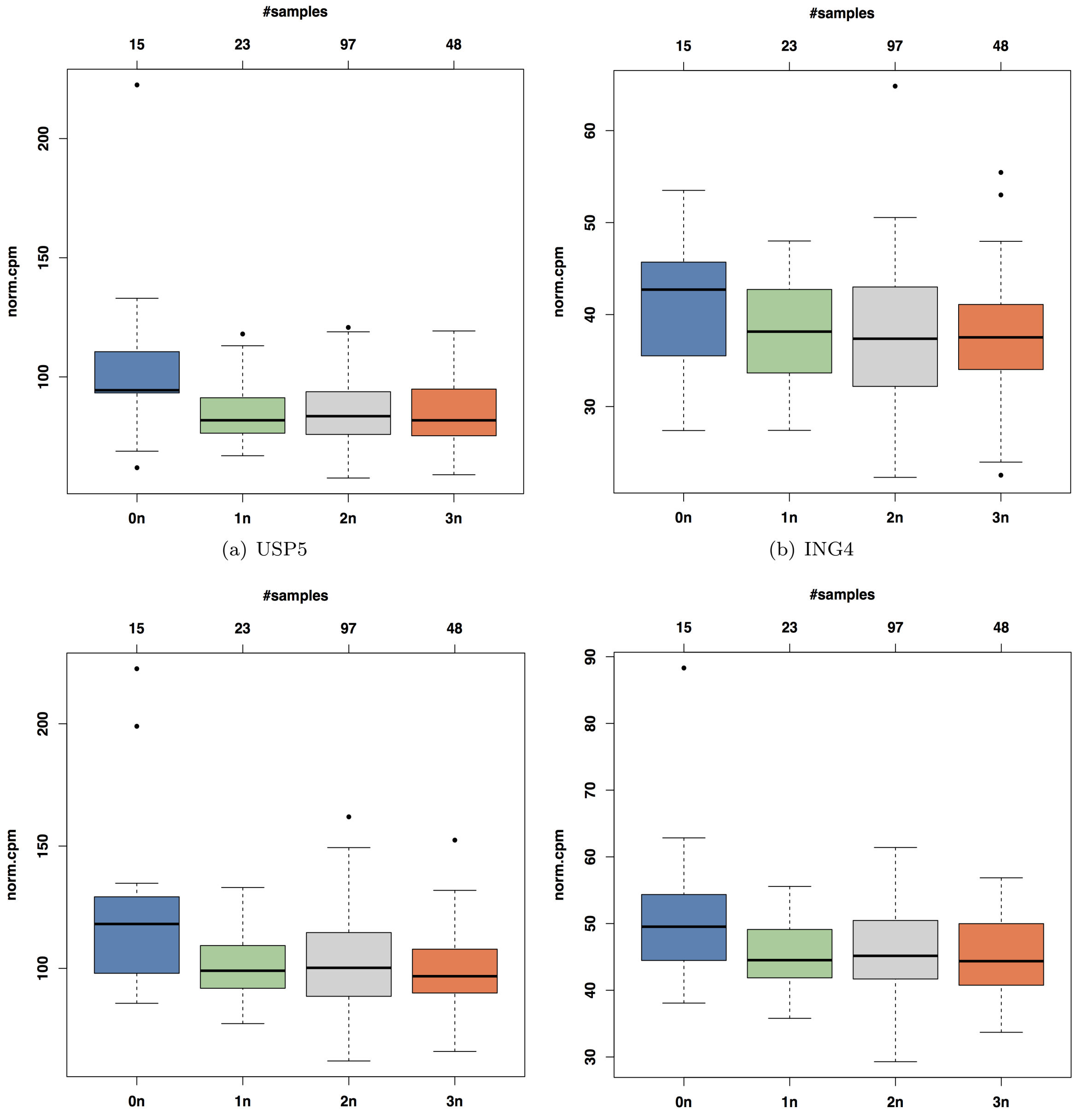

(a) USP5 (b) ING4

(c) MLF2 (d) ZNF384

Figure S9: Regulatory factors associated with R727.

2.5 R760

This a multi-allelic region on chromosome 5. This region displays high variability with the majority of the population carrying a non-diploid copy number, and only an exceptional small number of samples being in $2n$. Fine-mapping highlights a region at 117.55 $\pm$ 0.05 Mb, which is immediately upstream of Tetratricopeptide Repeat Domain 38 (*TTC38*, Figure S10a). *TTC38* interacts with thymidine kinase

1, *TK1*, which is involved in skeletal muscle growth [13]. Although generally lowly expressed, we found decreased expression of *TTC*38 (Figure S10b), whereas *TK1* expression was significantly increased in the exceptional $2n$ group.
On the other hand, we found expression in $2n$ samples increased for *ATXN10* and decreased for *FBLN1* (Figure S10c and d). *ATXN10* is involved in *Akt* signaling, a major regulatory pathway of skeletal muscle mass [14]. Concordantly, we also found *AKT1* expression increased for $2n$ samples.
FBLN1 interacts with *TCF7L2*, which regulates skeletal muscle tissue development by mediating WNT/β-catenin signaling [15]. Concordantly, we found muscle structure development processes enriched for the genes associated with R760. We also found ChIP-seq enrichment (when mapping R760 to human) of *TCF7L2* and significant down-regulation of *TCF7L2* in the exceptional $2n$ group.

2.6 R828

This is a bi-allelic region on chromosome 6 that contains TF *LCORL*. (Figure S11a). As depicted at the bottom of Figure S11a, `CNVnator` calls indicate that this is rather a copy number loss, which is consistent with the findings of Hou *et al.*, 2011 [1]. This region has been repeatedly found associated with body composition and feed efficiency [16]. Concordantly, we find anatomic development-related processes enriched in associated genes.
Whereas *LCORL* does not show substantial change in expression (Figure S11b), we found this region (when mapped to human) enriched for ChIP-seq binding of several TFs. This includes *SIX5*, a transcription factor involved in anatomic development, which we also found significantly up-regulated in expression in the $3n$ group. On the other hand, this also includes *SAP30*, a transcriptional repressor, which we found significantly down-regulated.

2.7 R1430

This is a multi-allelic region on chromosome 12. Fine-mapping highlights a regions at 1.175 $\pm$ 0.01 Mb, which locates 0.45 Mb upstream of *ENSBTAG00000035926* (reverse strand, Figure S12a). Ensembl consensus annotation of this gene is elongation factor 1-alpha (97% sequence similarity with cattle *EEF1A*), which we found down-regulated in the $1n$ group (Figure S12b). Interestingly, we also found the actual *EEF1A* (ENSBTAG00000014534) to be 2-fold down-regulated in the $1n$. *EEF1A* has been observed to exert pro-apoptotic activity in dying myotubes and accordingly found down-regulated in growing myotubes [17].

2.8 R1723-5

These three CNV regions locate proximal to each other between 10.3 Mb and 13.6 Mb within a gene desert on chromosome 15. *ENSBTAG00000009511* and *JRKL* locate 0.35 Mb and 0.48 b downstream of R1723-5, which we found up-regulated in the $3n$ group.
ENSBTAG00000009511 is a pseudogene of Protein Phosphatase 1 (*PP1*) Regulatory Inhibitor Subunit 14C, *PPP1R14C*. Interestingly, *PP1* is assumed to play an important role during myogenic differentiation as its inhibition affects *MYOD1* and *AP1* activity [18]. This is consistent with the enrichment of *AP1* binding and protein phosporylation-related processes among associated genes of R1723-5.
JRKL is a supposed nuclear regulatory protein, which shares similarity with *JRK*, a regulator of β-catenin transcriptional activity [19].

2.9 R1802

This is a tri-allelic region on chromosome 15. Fine-mapping highlights a region at 15.09 $\pm$ 0.02 Mb. The region locates 0.85 Mb downstream of *RCN1*, 0.81 Mb upstream of *LMO2*, and 0.41 Mb down-

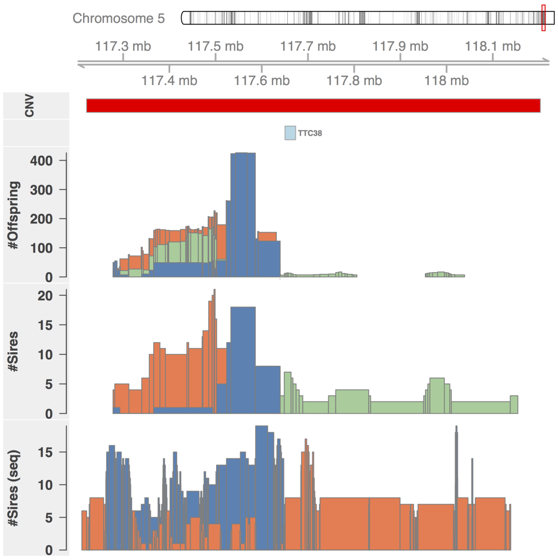

(a) Fine-mapping of R760

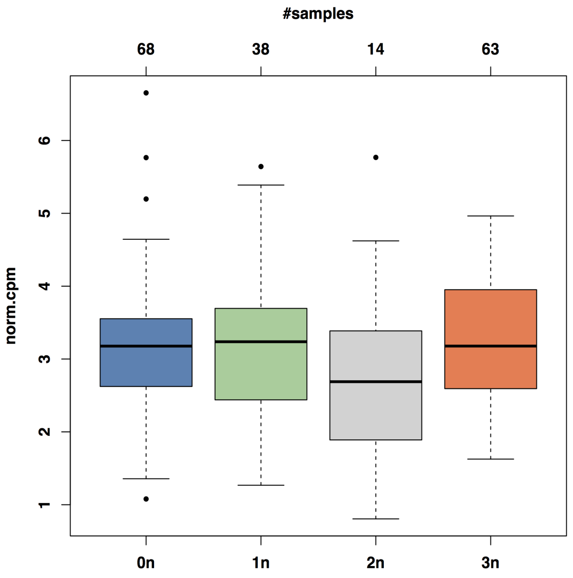

(b) *TTC38* expression

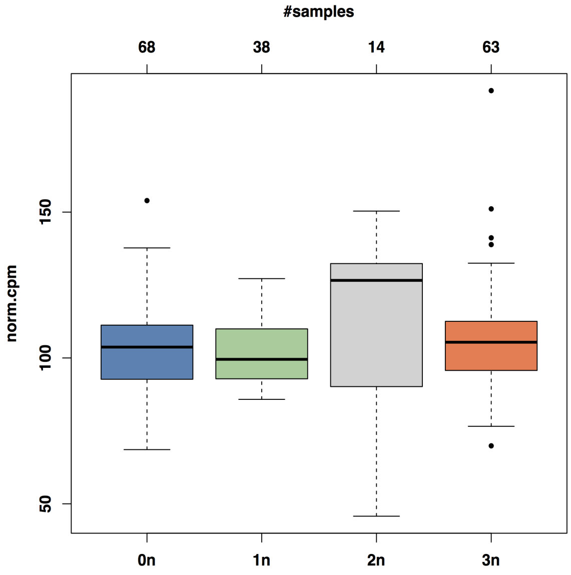

(c) *ATXN10* expression

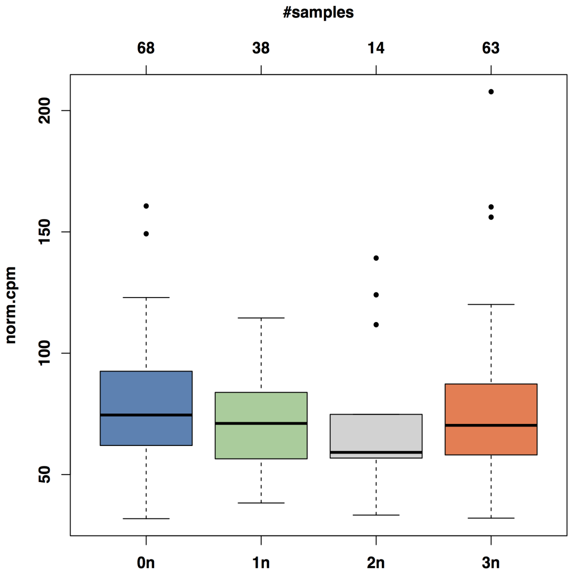

(d) *FBLN1* expression

Figure S10: **(a)** shows the location of R760 (CNV track in red) and the *TTC38* gene on chromosome 5. The data track below show the number of offspring that have been called by `PennCNV` to contain a one copy gain (3n, orange) in R162. The data track at the bottom shows corresponding CNV-seq calls from `CNVnator`. The boxplot in **(b)** shows the expression of *ATXN10* (y-axis, normalized counts per million reads mapped) stratified by CN state (x-axis). The number of samples in each CN group is indicated on top of the plot.

stream of *EIF3M*. We found these three genes down-regulated in the 3n group. Reticulocalbin 1, *RCN1*, is a calcineurin inhibitor [20], where calcineurin regulates skeletal muscle metabolism via coordinated

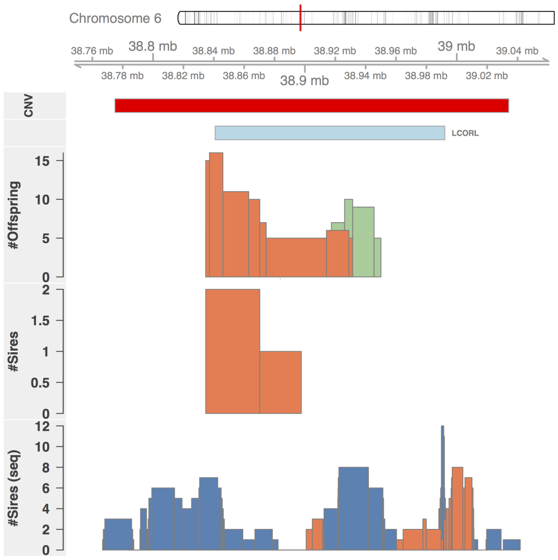

(a) Fine-mapping of R828

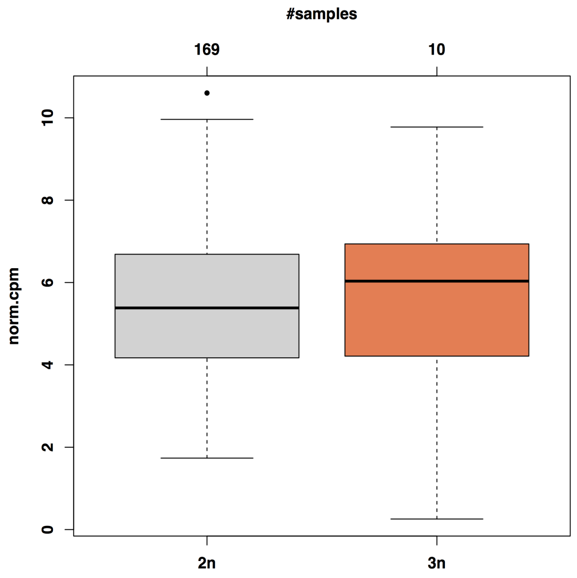

(b) *LCORL* expression

Figure S11: (a) shows the location of R828 (CNV track in red) and the *LCORL* gene on chromosome 6. The two data tracks below show the number of offspring and sires, respectively, that have been called by `PennCNV` to contain a one copy loss ($1n$, green) or gain (3n, orange) in R828. The third data track at the bottom shows corresponding CNV-seq calls from `CNVnator`. The boxplot in **(b)** shows the expression of *LCORL* (y-axis, normalized counts per million reads mapped) stratified by CN state (x-axis). The number of samples in each CN group is indicated on top of the plot.

changes in gene expression [21]. LIM Domain Only 2, *LMO2*, is a modulator of *TAL1* transcriptional activity [22], where *TAL1* regulates myogenic differentiation in skeletal muscle [23]. *EIF3M* encodes subunit M of eukaryotic translation initiation factor 3, which is of importance as EIF3 subunits have been previously found involved in skeletal muscle processes. For instance, *EIF3F* is a central regulator of atrophy/hypertrophy in skeletal muscle [24].

2.10 R1869

This is a bi-allelic region on chromosome 16. Fine-mapping highlights a region at 15.09 ± 0.02 Mb. The region locates 1.2 Mb upstream (reverse strand) of a Regulator of G-protein signaling (RGS) gene cluster. This includes *RGS2*, which we found upregulated in the $1n$ group. Interestingly, *RGS2* coincides with another *7SK* RNA (see R721). Another coinciding gene, *CDC73*, which we found down-regulated in the $1n$ group, is a component of the *PAF1* complex (*PAF1C*). *CDC73* connects *PAF1C* with Wnt signaling and myogenesis.

2.11 R2440

This is a bi-allelic region on chromosome 25. Fine-mapping highlights a region at 37.13 ± 0.01 Mb. The region locates within a gene cluster, which contains several muscle-relevant regulatory factors. This includes *TSC22D4*, *GNB2*, and *GIGYF1*, for which we found expression to be associated with CN in R2440.
TSC22 Domain Family Member 4, *TSC22D4*, is a member of the TSC22 domain family of leucine zipper

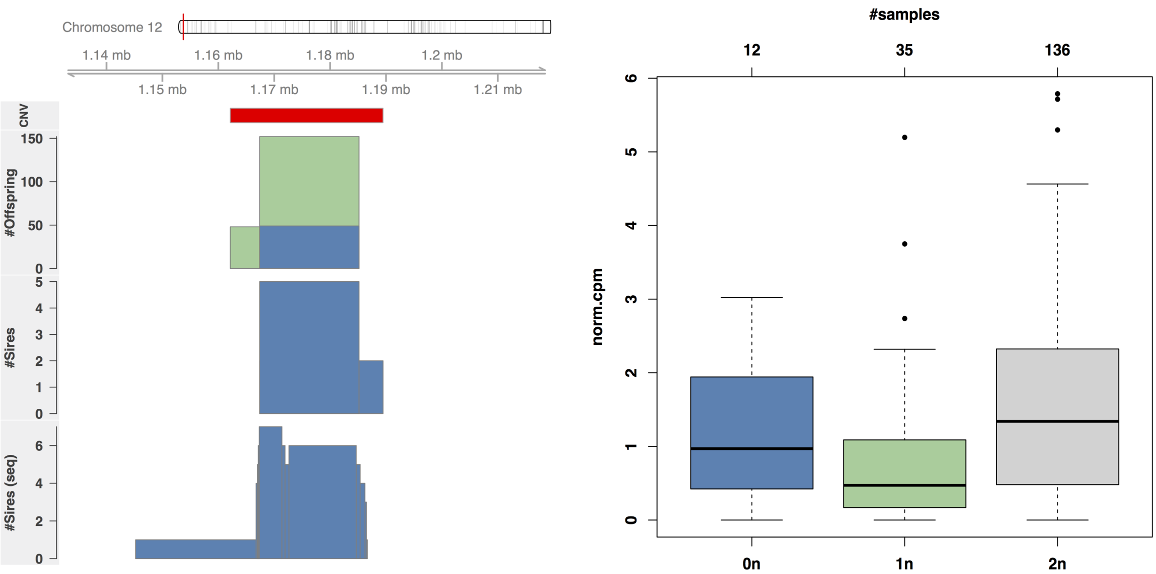

(a) Fine-mapping of R1430

(b) *EEF1A1** expression

Figure S12: (a) shows the location of R1430 (CNV track in red) and the *EEF1A1** gene on chromosome 12. The data track below show the number of offspring that have been called by `PennCNV` to contain a one copy gain (3n, orange) in R1430. The data track at the bottom shows corresponding CNV-seq calls from `CNVnator`. The boxplot in **(b)** shows the expression of *EEF1A1** (y-axis, normalized counts per million reads mapped) stratified by CN state (x-axis). The number of samples in each CN group is indicated on top of the plot.

transcriptional regulators. Its paralog *TSC22D3* has been found to suppress *AP1* and *NFKB1* DNA-binding activities, inhibit myogenic differentiation and mediate anti-myogenic effects of glucocorticoids by binding and regulating *MYOD1* and *HDAC1* transcriptional activity resulting in reduced expression of *MYOG* [25].
G Protein Subunit Beta 2, *GNB2*, is of importance as G protein signaling is a key regulatory process of skeletal muscle myogenesis [26].
The GRB10 Interacting GYF Protein 1, *GIGYF1*, has been reported to act cooperatively with *GRB10* in regulating tyrosine kinase receptor signaling. *GRB10* regulates muscle cell proliferation and the development of fiber number in skeletal muscle [27].
Additional transcriptional regulators in the vicinity of R2440 include another subunit of the COP9 signalosome complex (COPS6), several zinc fingers (ZKSCAN1, ZSCAN21, ZNF394, ZNF655, ZNF789) and microRNAs (MIR25, MIR93, MIR106b).

2.12 R2461

This is a bi-allelic region on chromosome 26. Fine-mapping highlights a region at 2.955 $\pm$ 0.01 Mb. The region locates 2.1 Mb downstream of the inositol polyphosphate multikinase, *IPMK*, and 1 Mb upstream of *ENSBTAG00000048299*, which corresponds to *MIR3924* in human. Although in general lowly expressed, we found *IPMK* expression decreased in the $3n$ sample group. *IPMK* promotes myogenic differentiation since its overexpression results in up-regulation of several myogenic markers and causes an increase in β-catenin translocation [28]. *MIR3924* target genes include *COPS8*, *ETS1*, *MYRF*, and *TCF7L2*.

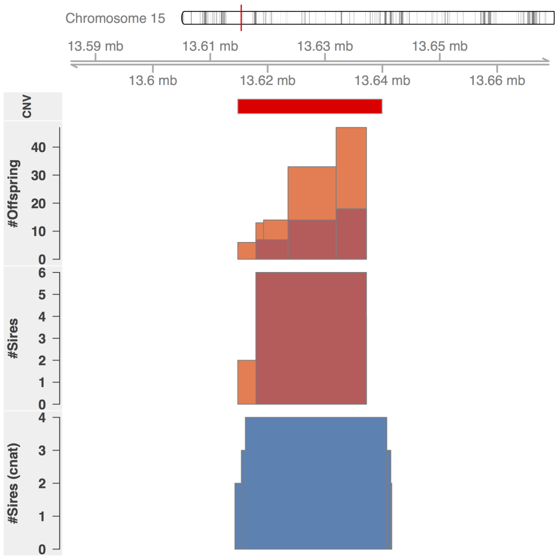

(a) Fine-mapping of R1725

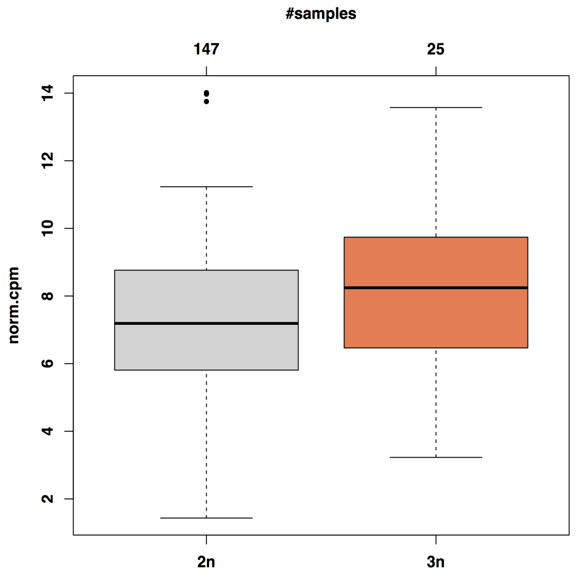

(b) *PPP1R14C** expression

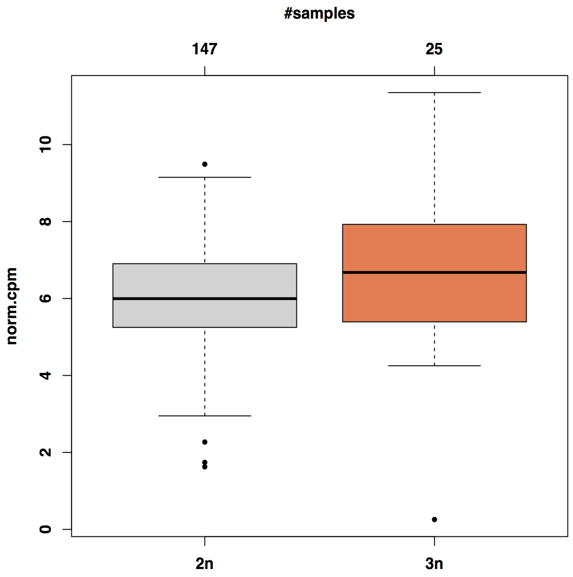

(c) *JRKL* expression

Figure S13: (a) shows the location of R1725 (CNV track in red) on chromosome 15. The two data tracks below show the number of offspring and sires, respectively, that have been called by `PennCNV` as a one copy ($3n$, orange) or two copy gain ($4n$, darkred) in R1725. The third data track at the bottom shows corresponding CNV-seq calls from `CNVnator` (deletions in blue). The boxplots **(b,c)** show the expression of the proximal located *ENSBTAG00000009511/PPP1R14C** and *JRKL* (y-axis, normalized counts per million reads mapped) stratified by CN state (x-axis) in R1725. The number of samples in each CN group is indicated on top of the plot.

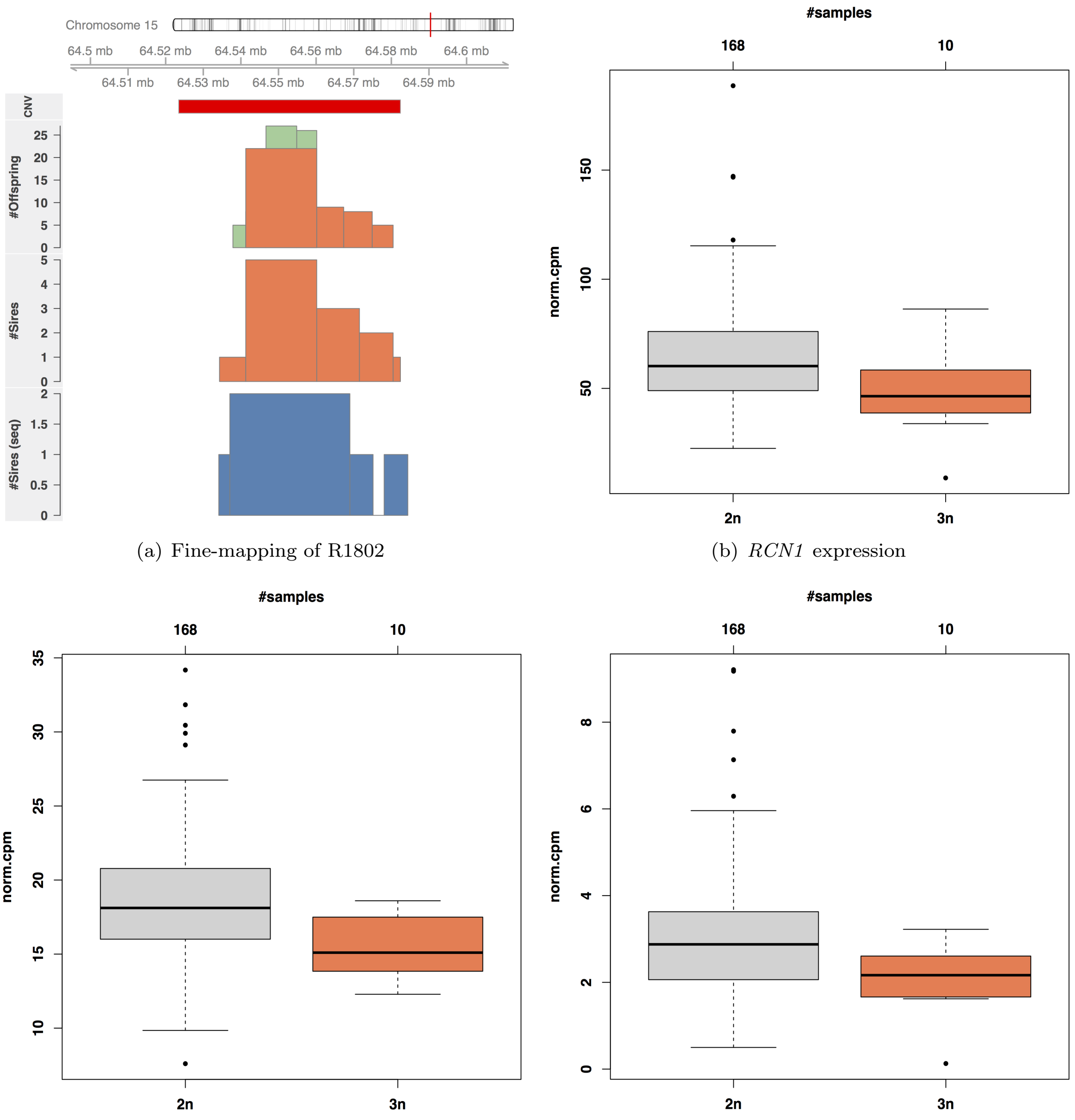

(a) Fine-mapping of R1802

(b) *RCN1* expression

(c) *LMO2* expression

(d) *EIF3M* expression

Figure S14: (a) shows the location of R1802 (CNV track in red) on chromosome 15. The two data tracks below show the number of offspring and sires, respectively, that have been called by `PennCNV` as a one copy loss ($1n$, green) or gain ($3n$, orange) in R1802. The third data track at the bottom shows corresponding CNV-seq calls from `CNVnator` (deletions in blue). The boxplots **(b-d)** show the expression of the proximal located *RCN1*, *LMO2*, and *EIF3M* (y-axis, normalized counts per million reads mapped) stratified by CN state (x-axis) in R1802. The number of samples in each CN group is indicated on top of the plot.

3 Phenotype effects

References

[1] Hou, Y., Liu, G.E., Bickhart, D.M., Cardone, M.F., Wang, K., Kim, E., Matukumalli, L.K., Ventura,

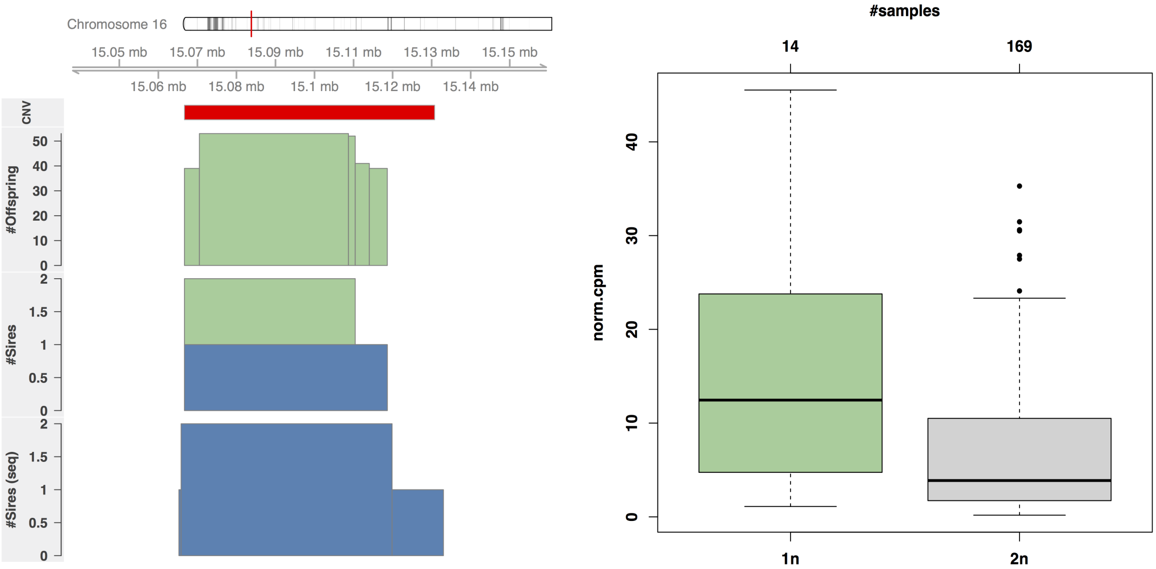

(a) Fine-mapping of R1869 (b) *RGS2* expression

Figure S15: **(a)** shows the location of R1869 (CNV track in red) on chromosome 16. The two data tracks below show the number of offspring and sires, respectively, that have been called by `PennCNV` to contain a complete (0n, blue) or partial (1n, green) deletion in R1869. The third data track at the bottom shows corresponding CNV-seq calls from `CNVnator` (deletions in blue). The boxplot in **(b)** shows the expression of *RGS2* (y-axis, normalized counts per million reads mapped) stratified by CN state (x-axis) in R1869. The number of samples in each CN group is indicated on top of the plot.

M., Song, J., VanRaden, P.M., *et al.*: Genomic characteristics of cattle copy number variations. BMC Genomics **12**(1), 127 (2011)

[2] Suzuki, T., Ueda, A., Kobayashi, N., Yang, J., Tomaru, K., *et al.*: Proteasome-dependent degradation of alpha-catenin is regulated by interaction with ARMc8alpha. Biochem J **411(3)**, 581–91 (2008)

[3] Hwang, S.G., Yu, S.S., Ryu, J.H., Jeon, H.B., Yoo, Y.J., *et al.*: Regulation of beta-catenin signaling and maintenance of chondrocyte differentiation by ubiquitin-independent proteasomal degradation of alpha-catenin. J Biol Chem **280(13)**, 12758–65 (2005)

[4] Suzuki, A., Scruggs, A., Iwata, J.: The temporal specific role of WNT/b-catenin signaling during myogenesis. J Nat Sci **1(8)**, 143 (2015)

[5] Diribarne, G., Bensaude, O.: 7SK RNA, a non-coding RNA regulating P-TEFb, a general transcription factor. RNA Biol **6(2)**, 122–8 (2009)

[6] Simone, C., Stiegler, P., Bagella, L., Pucci, B., Bellan, C., *et al.*: Activation of MyoD-dependent transcription by cdk9/cyclin T2. Oncogene **21(26)**, 4137–48 (2002)

[7] De Angelis, L., Balasubramanian, S., Berghella, L.: Akt-mediated phosphorylation controls the activity of the Y-box protein MSY3 in skeletal muscle. Skelet Muscle **5**, 18 (2015)

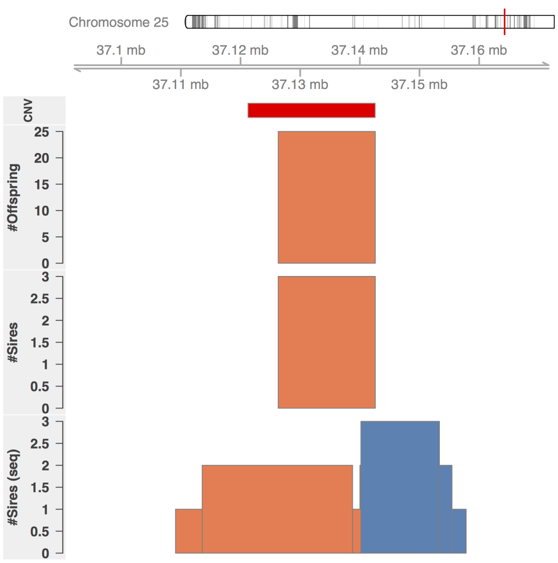

(a) Fine-mapping of R2440

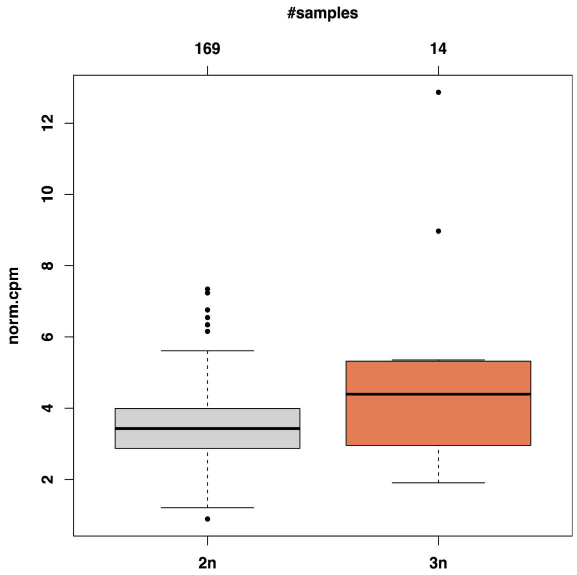

(b) *TSC22D4* expression

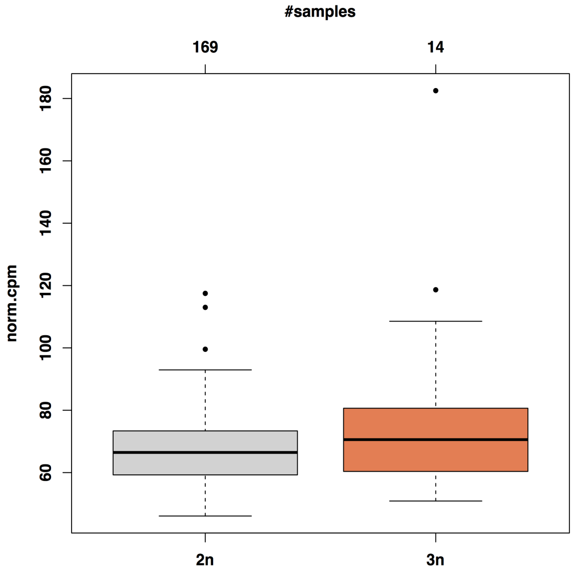

(c) *GNB2*

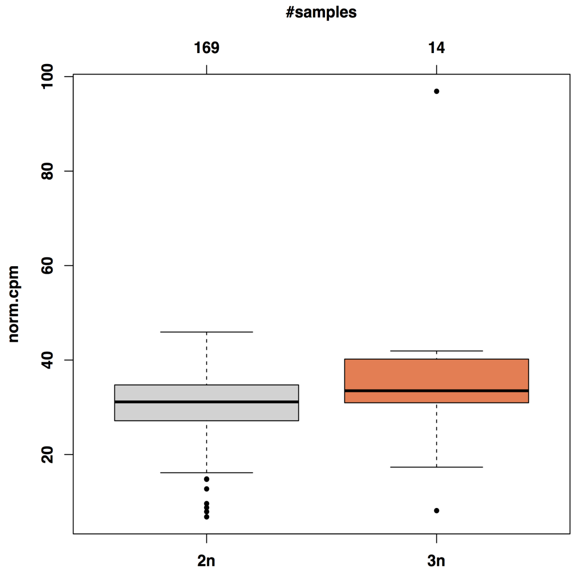

(d) *GIGYF1*

Figure S16: (a) shows the location of R2440 (CNV track in red) on chromosome 25. The data tracks below show the number of offspring and sires, respectively, that have been called by `PennCNV` to contain a one copy gain ($3n$, orange) in R2440. The data track at the bottom shows corresponding CNV-seq calls from `CNVnator` (deletions in blue and duplications in orange). The boxplot in **(b)** shows the expression of the 0.4 Mb upstream located *TSC22D4* (y-axis, normalized counts per million reads mapped) stratified by CN state (x-axis) in R2440. The number of samples in each CN group is indicated on top of the plot.

[8] Sun, L., Liu, L., Yang, X.J., Wu, Z.: Akt binds prohibitin 2 and relieves its repression of MyoD and

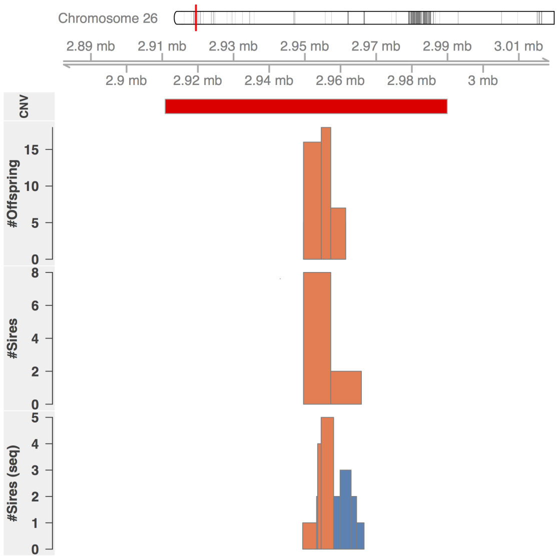

(a) Fine-mapping of R2461

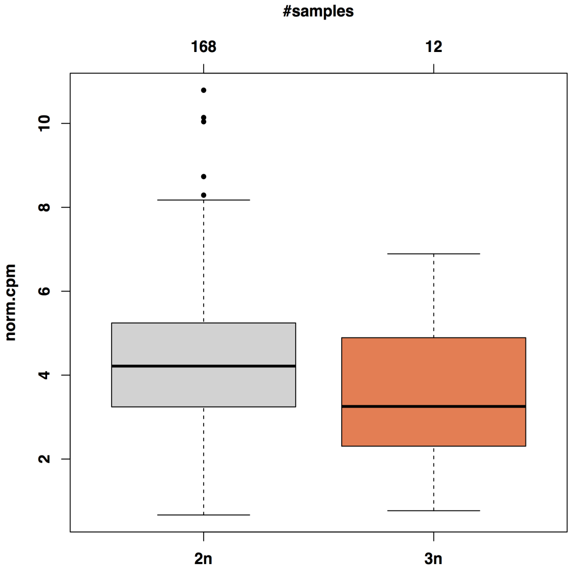

(b) *IPMK* expression

Figure S17: **(a)** shows the location of R2461 (CNV track in red) on chromosome 26. The data tracks below show the number of offspring and sires, respectively, that have been called by `PennCNV` to contain a one copy gain ($3n$, orange) in R2461. The data track at the bottom shows corresponding CNV-seq calls from `CNVnator` (deletions in blue and duplications in orange). The boxplot in **(b)** shows the expression of the 2.1 Mb upstream located *IPMK* (y-axis, normalized counts per million reads mapped) stratified by CN state in R2461 (x-axis). The number of samples in each CN group is indicated on top of the plot.

muscle differentiation. J Cell Sci **117(14)**, 3021–9 (2004)

[9] Yang, Z.J., Broz, D.K., Noderer, W.L., Ferreira, J.P., Overton, K.W., *et al.*: p53 suppresses muscle differentiation at the myogenin step in response to genotoxic stress. Cell Death Differ **22(4)**, 560–73 (2015)

[10] Giacinti, C., Bagella, L., Puri, P.L., Giordano, A., Simone, C.: MyoD recruits the cdk9/cyclin T2 complex on myogenic-genes regulatory regions. J Cell Physiol **206(3)**, 807–13 (2006)

[11] Spangenburg, E.E., Chakravarthy, M.V., Booth, F.W.: p27Kip1: a key regulator of skeletal muscle satellite cell proliferation. Clin Orthop Relat Res **403**, 221–7 (2002)

[12] Chen, X., Li, Y.: Role of matrix metalloproteinases in skeletal muscle: migration, differentiation, regeneration and fibrosis. Cell Adh Migr **3(4)**, 337–41 (2009)

[13] Gross, M.K., Merrill, G.F.: Thymidine kinase synthesis is repressed in nonreplicating muscle cells by a translational mechanism that does not affect the polysomal distribution of thymidine kinase mRNA. PNAS **86(13)**, 4987–91 (1989)

[14] Egerman, M.A., Glass, D.J.: Signaling pathways controlling skeletal muscle mass. Crit Rev Biochem Mol Biol **49(1)**, 59–68 (2014)

[15] Hou, N., Ye, B., Li, X., Margulies, K.B., Xu, H., *et al.*: Transcription Factor 7-like 2 Mediates Canonical Wnt/beta-Catenin Signaling and c-Myc Upregulation in Heart Failure. Circ Heart Fail **9(6)**, 003010 (2016)

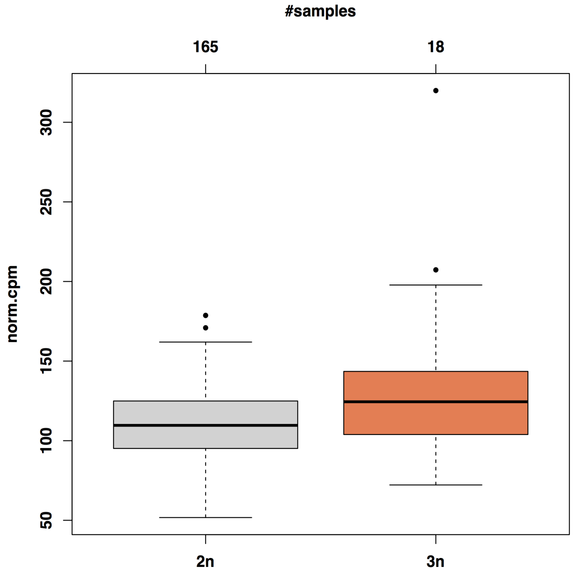

(a) R129: *MYH14* expression

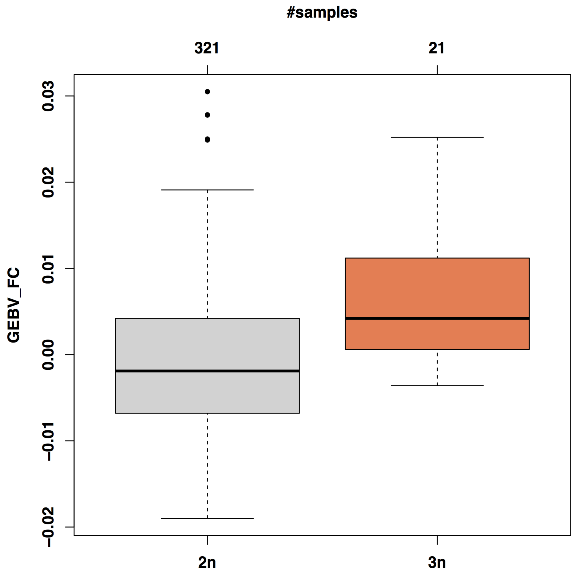

(b) R129: feed conversion

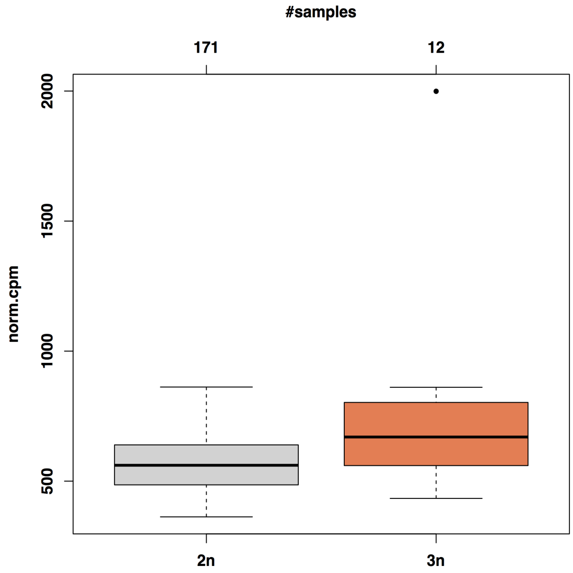

(c) R162: *MYLK** expression

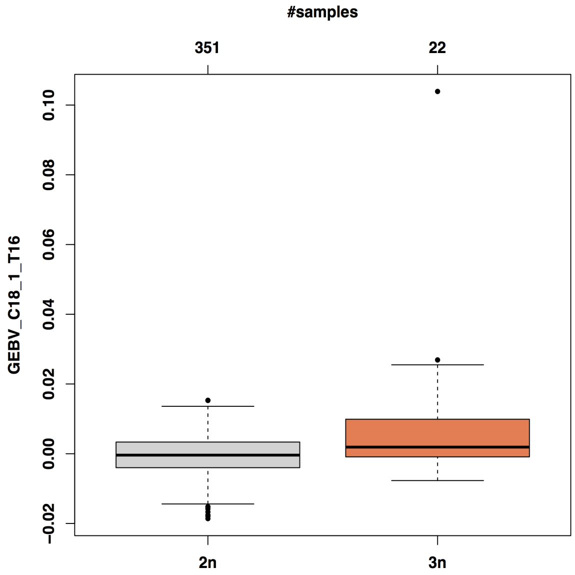

(d) R162: C18-1-T16

Figure S18: Multi-level associations detected at the interface of copy number variation, gene expression and phenotype. **(a)** shows the expression of *MYH14* (y-axis, normalized counts per million reads mapped) stratified by CN state in R129 (x-axis). Myosin heavy chain 14 (*MYH14*) is chosen here as a representative of the various R129-associated genes that display similar expression patterns. **(b)** shows genomic estimated breeding values for feed conversion (ratio of dry matter intake and average daily gain, y-axis) stratified by CN state in R129 (x-axis). Analogously, **(c)** and **(d)** show expression of ENSBTAG00000040028 (ENSEMBL consensus annotation: myosin light chain kinase obscurin) and genomic estimated breeding values for $C18-1-T16$ concentration (Trans-16 Octadecenoic acid, a monounsaturated fatty acid) stratified by CN state in R162. The number of samples in each CN group is indicated on top of each boxplot.

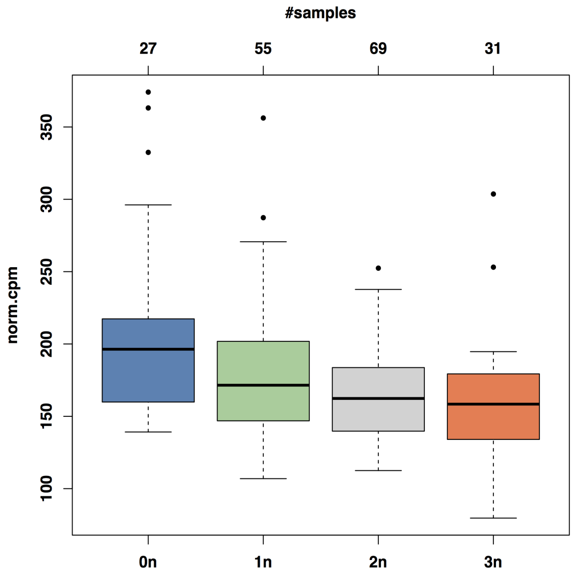

(a) R721: *MPC1* expression

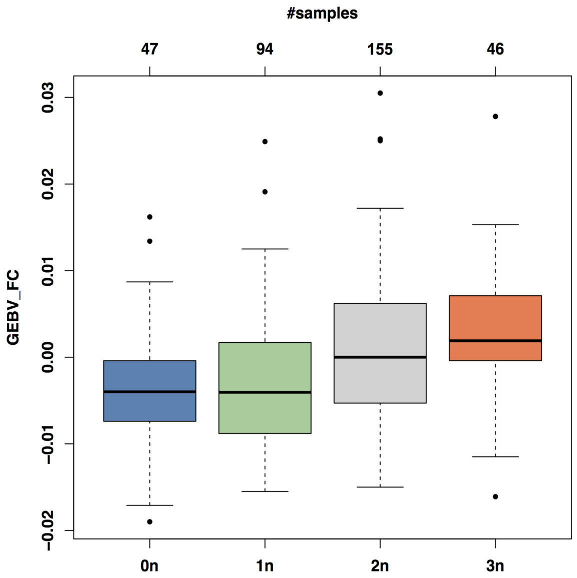

(b) R721: feed conversion

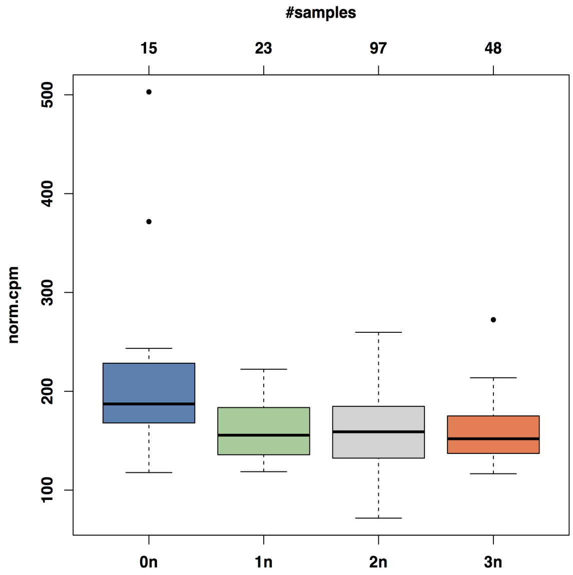

(c) R727: *SCGA* expression

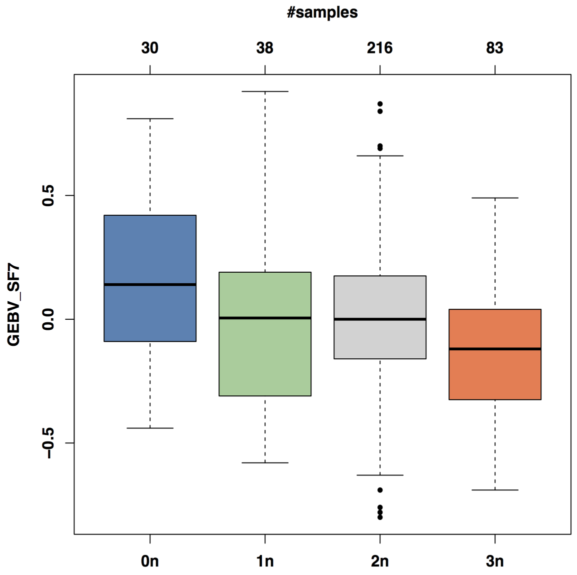

(d) R727: shear force (7 days)

Figure S19: Multi-level associations detected at the interface of copy number variation, gene expression and phenotype. Analogous to Figure S18, **(a)** and **(b)** show the expression of Mitochondrial pyruvate carrier 1 (*MPC1*) and genomic estimated breeding values for feed conversion stratified by CN state in R721. Likewise, **(c)** and **(d)** show expression of Sarcogylcan alpha (*SCGA*) and genomic estimated breeding values for shear force (measured 7 days after slaughter) stratified by CN state in R727.

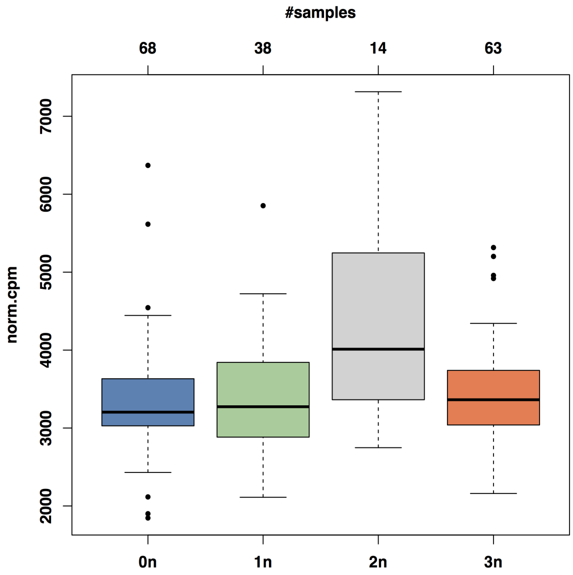

(a) R760: *YBX3* expression

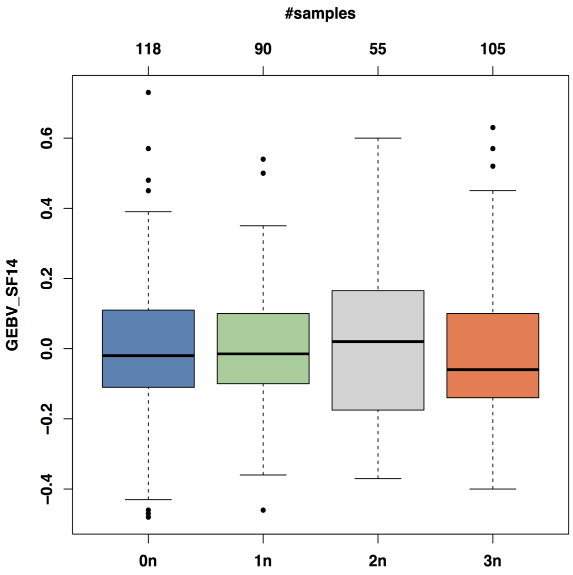

(b) R760: shear force (14 days)

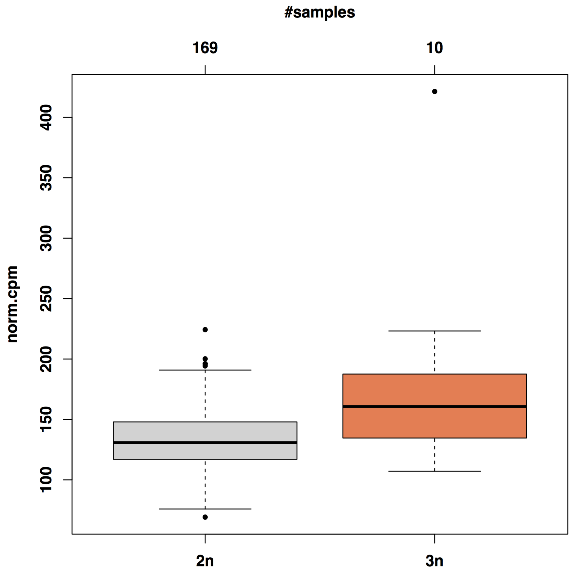

(c) R828: *MEF2D* expression

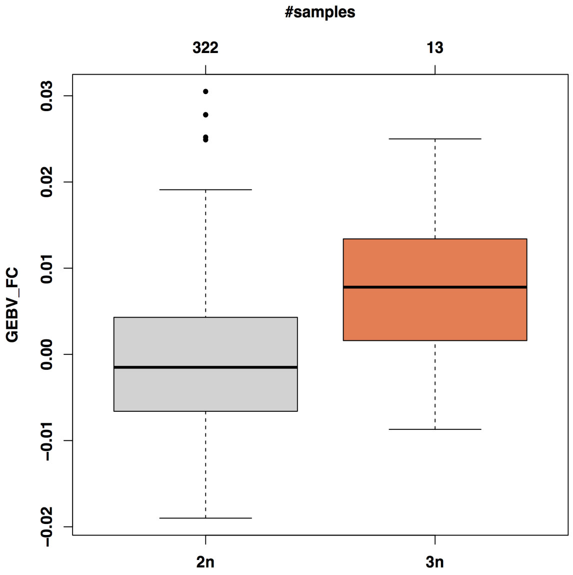

(d) R828: feed conversion

Figure S20: Multi-level associations detected at the interface of copy number variation, gene expression and phenotype. Analogous to Figure S18, **(a)** and **(b)** show the expression of Y-box binding protein 3 (*YBX3*) and genomic estimated breeding values for shear force (measured 14 days after slaughter) stratified by CN state in R760. Likewise, **(c)** and **(d)** show expression of Myocyte enhancer factor 2D (*MEF2D*) and genomic estimated breeding values for feed conversion stratified by CN state in R828.

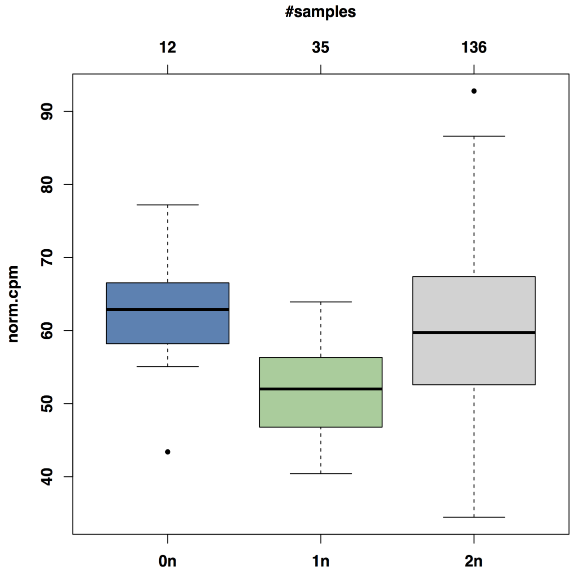

(a) R1430: *KMT5A* expression

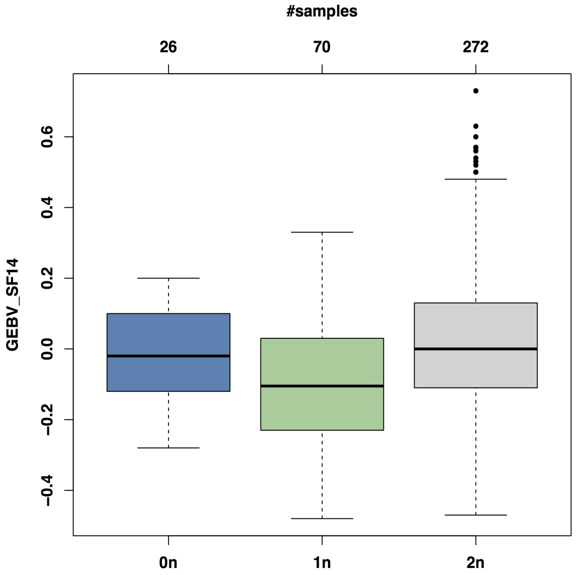

(b) R1430: shear force (14 days)

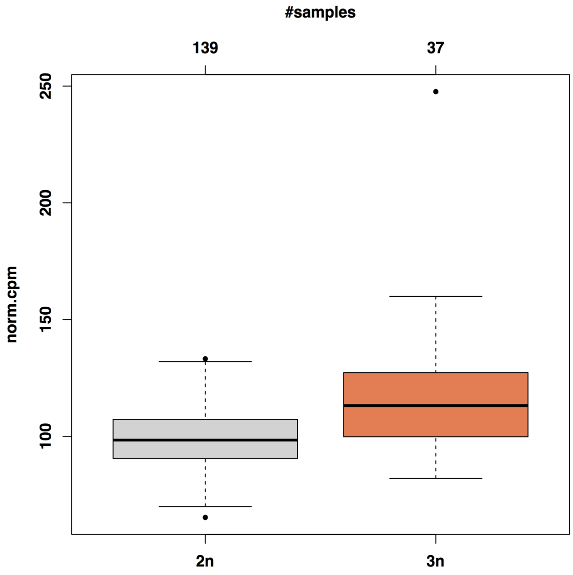

(c) R1723: *MTMR3* expression

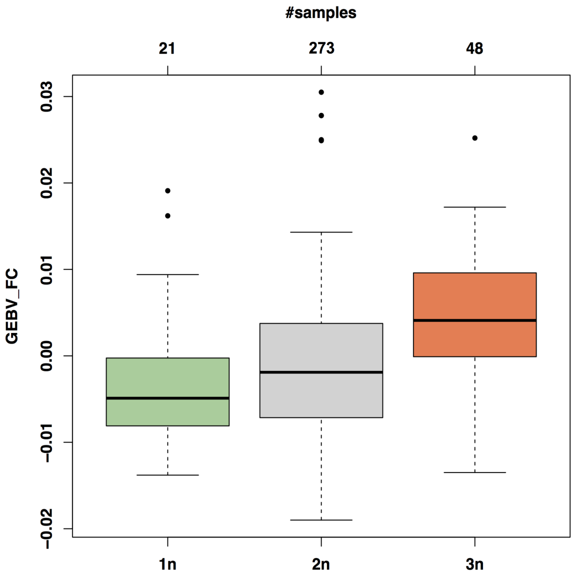

(d) R1723: feed conversion

Figure S21: Multi-level associations detected at the interface of copy number variation, gene expression and phenotype. Analogous to Figure S18, **(a)** and **(b)** show the expression of Lysine methyltransferase 5A (*KMT5A*) and genomic estimated breeding values for shear force (measured 14 days after slaughter) stratified by CN state in R1430. Likewise, **(c)** and **(d)** show expression of Myotubularin related protein 3 (*MTMR3*) and genomic estimated breeding values for feed conversion stratified by CN state in R1723.

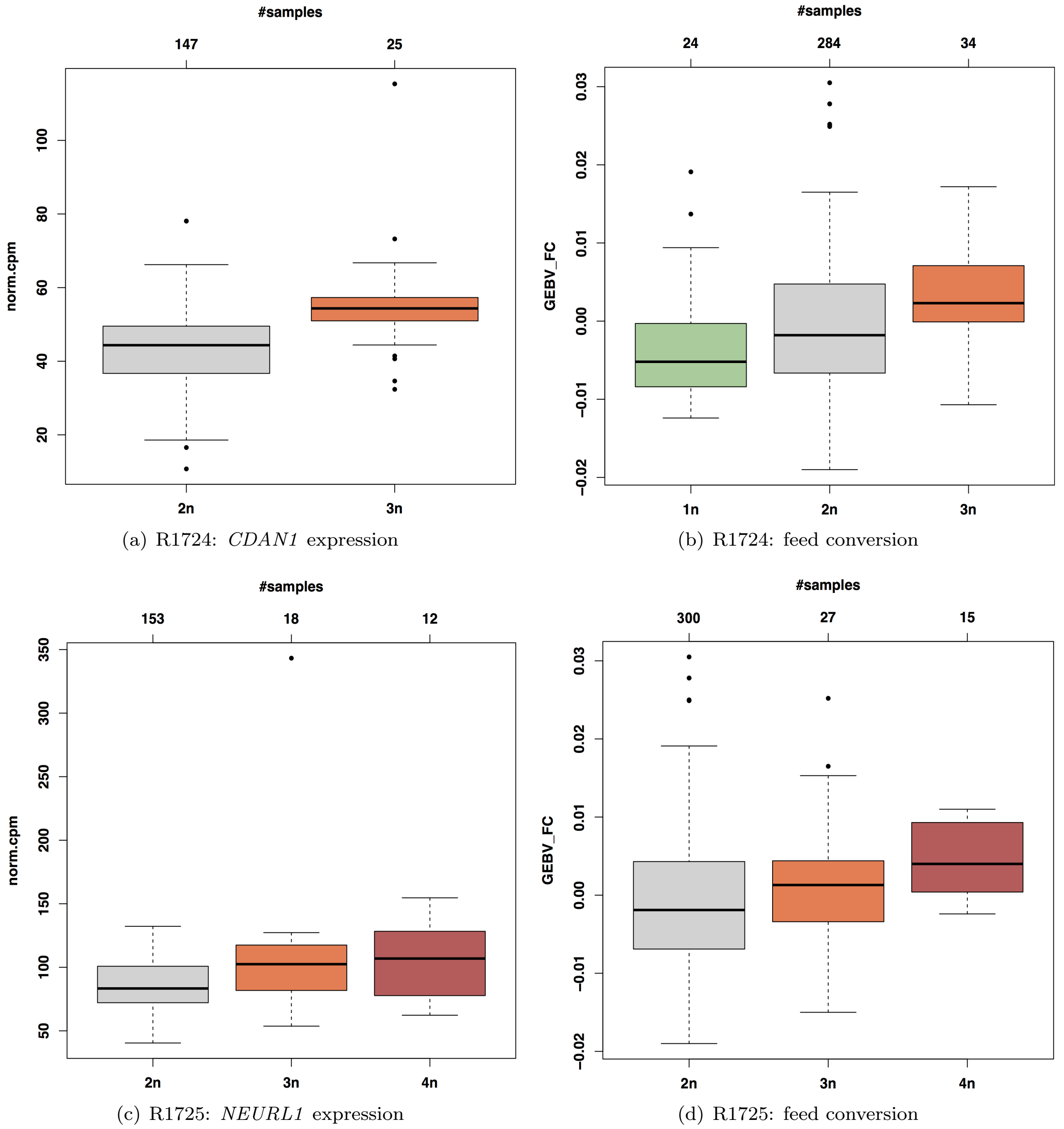

(a) R1724: *CDAN1* expression

(b) R1724: feed conversion

(c) R1725: *NEURL1* expression

(d) R1725: feed conversion

Figure S22: Multi-level associations detected at the interface of copy number variation, gene expression and phenotype. Analogous to Figure S18, **(a)** and **(b)** show the expression of Codanin 1 (*CDAN1*) and genomic estimated breeding values for feed conversion stratified by CN state in R1724. Likewise, **(c)** and **(d)** show expression of Neuralized E3 ubiquitin protein ligase 1 (*NEURL1*) and genomic estimated breeding values for feed conversion stratified by CN state in R1725.

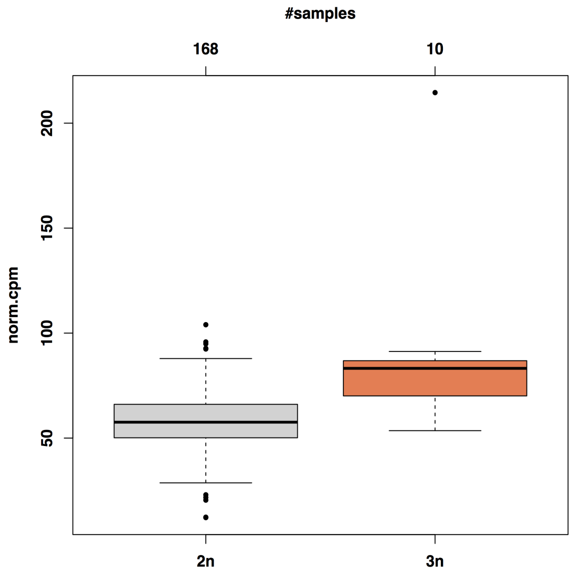

(a) R1802: *MLLT6* expression

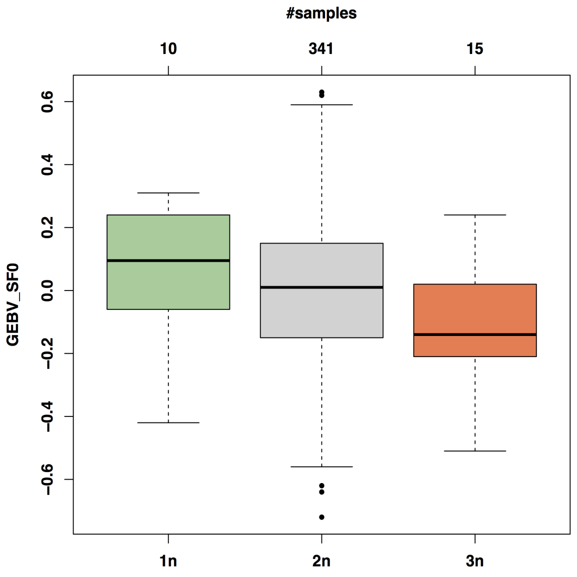

(b) R1802: shear force (after slaughter)

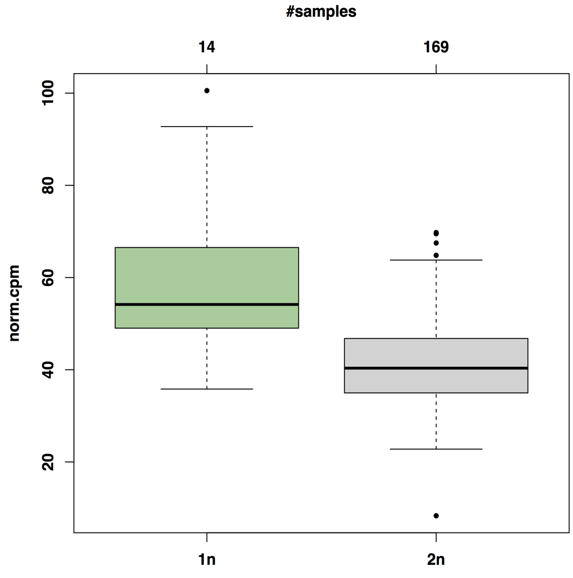

(c) R1869: *CYB5A* expression

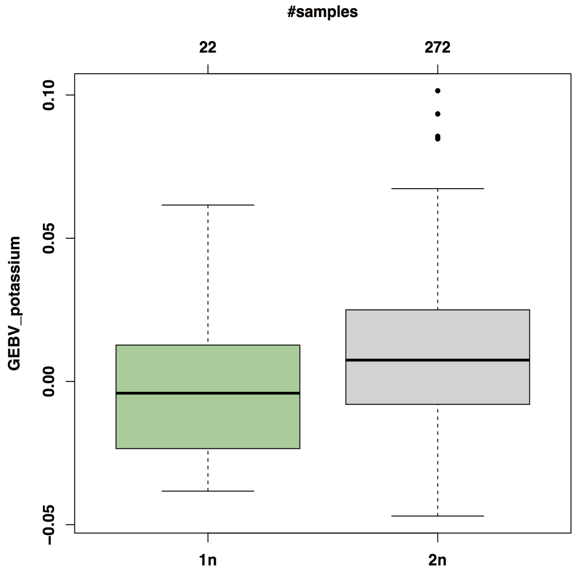

(d) R1869: potassium

Figure S23: Multi-level associations detected at the interface of copy number variation, gene expression and phenotype. Analogous to Figure S18, **(a)** and **(b)** show the expression of *MLLT6* and genomic estimated breeding values for shear force (after slaughter) stratified by CN state in R1802. Likewise, **(c)** and **(d)** show expression of Cytochrome b5 type A (*CYB5A*) and genomic estimated breeding values for potassium concentration stratified by CN state in R1869.

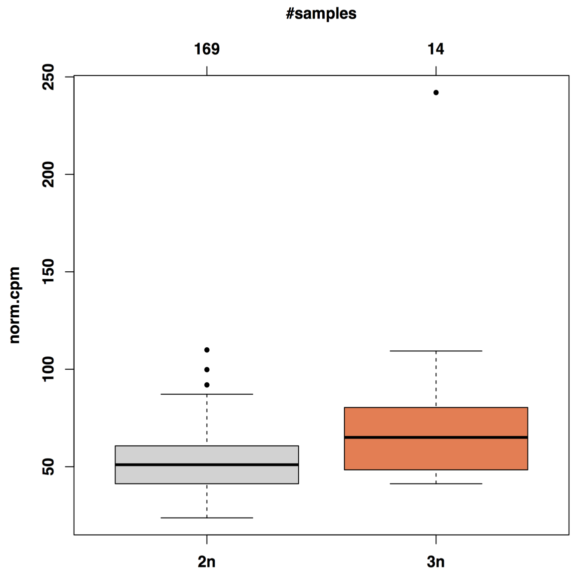

(a) R2440: *RBM38* expression

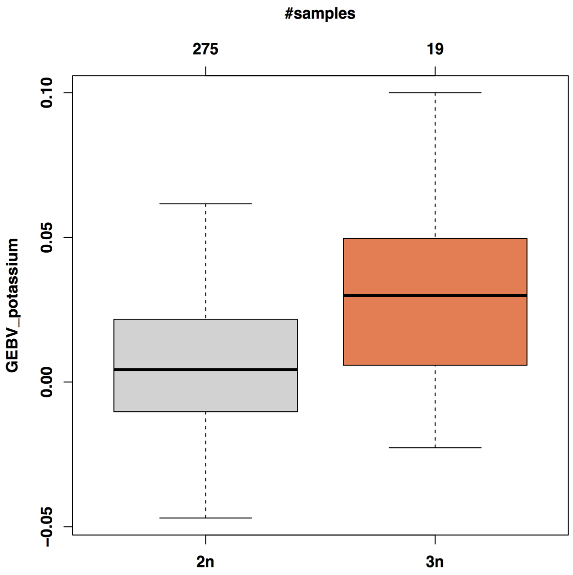

(b) R2440: potassium

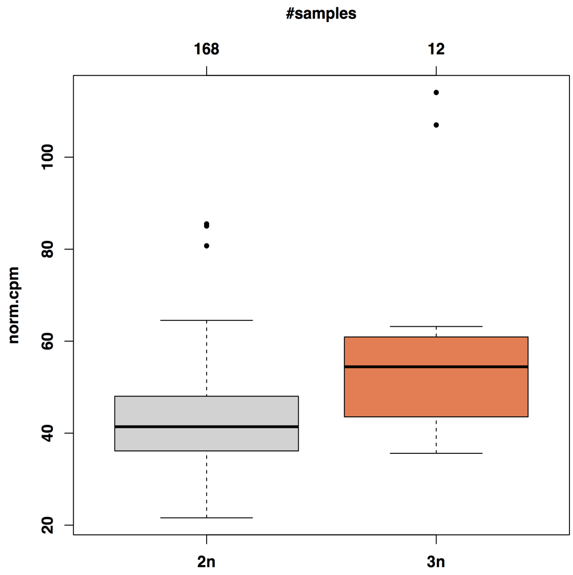

(c) R2461: *INSYN1* expression

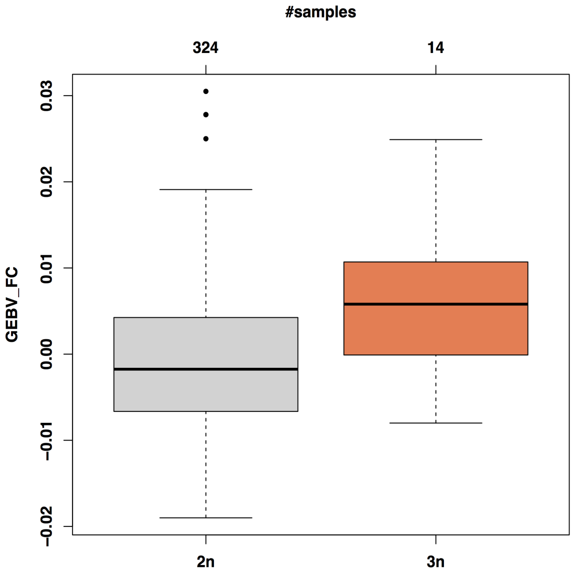

(d) R2461: feed conversion

Figure S24: Multi-level associations detected at the interface of copy number variation, gene expression and phenotype. Analogous to Figure S18, **(a)** and **(b)** show the expression of RNA binding motif protein 38 (*RBM38*) and genomic estimated breeding values for potassium concentration stratified by CN state in R2440. Likewise, **(c)** and **(d)** show expression of Inhibitory Synaptic Protein 1 (*INSYN1*) and genomic estimated breeding values for feed conversion stratified by CN state in R2461.

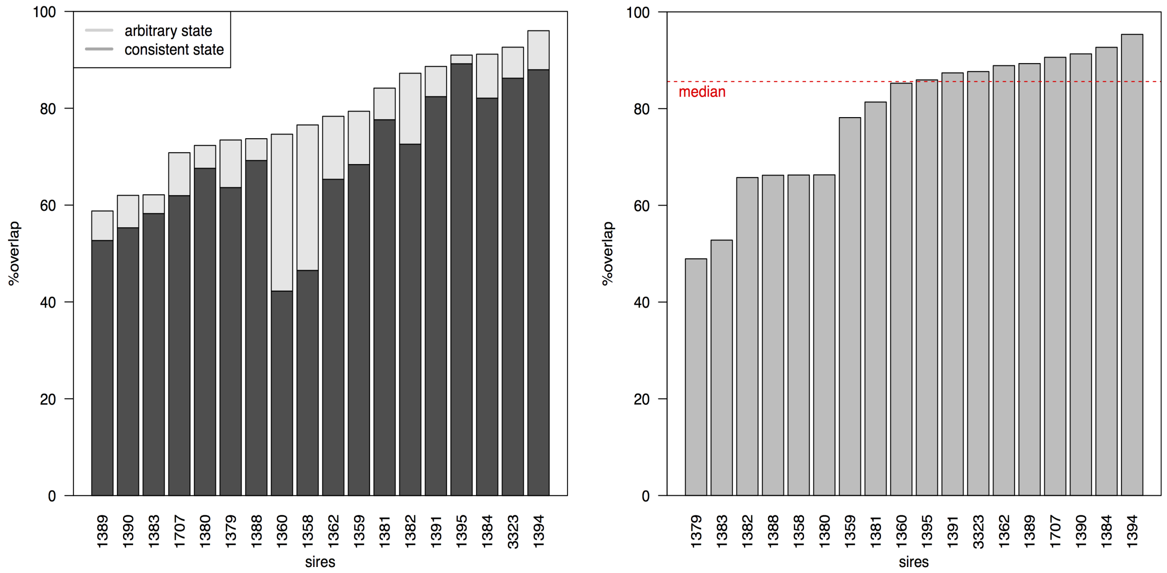

(a) overall

(b) deletions only

Figure S25: Concordance of snp- and seq-based CNV calls. (a) shows for each of the 18 sequenced sires (x-axis) the percentage of base pairs called as CNV in the snp-based approch (`PennCNV`) that were also called as CNV in the seq-based approach (`SpeedSeq` = `CNVnator` + `Lumpy`). The height of the grey bars indicate the concordance when not taking CNV state (deletion/duplication) into account, whereas black bars show concordance when requiring states to be consistent. Analogously, **(b)** shows the evaluation when only considering deletions (median concordance indicated by the red dashed line).

[16] Takasuga, A.: PLAG1 and NCAPG-LCORL in livestock. Anim Sci J **87(2)**, 159–67 (2016)

[17] Ruest, L.B., Marcotte, R., Wang, E.: Peptide elongation factor eEF1A-2/S1 expression in cultured differentiated myotubes and its protective effect against caspase-3-mediated apoptosis. J Biol Chem **277(7)**, 5418–25 (2002)

[18] Park, K., Chung, M., Kim, S.J.: Inhibition of myogenesis by okadaic acid, an inhibitor of protein phosphatases, 1 and 2A, correlates with the induction of AP1. J Biol Chem **267(15)**, 10810–5 (1992)

[19] Benchabane, H., Xin, N., Tian, A., Hafler, B.P., Nguyen, K., *et al.*: Jerky/Earthbound facilitates cell-specific Wnt/Wingless signalling by modulating b-catenin-TCF activity. EMBO J **30(8)**, 1444–58 (2011)

[20] Kishi, T., Ikeda, A., Nagao, R., Koyama, N.: The SCFCdc4 ubiquitin ligase regulates calcineurin signaling through degradation of phosphorylated Rcn1, an inhibitor of calcineurin. PNAS **104(44)**, 17418–23 (2007)

[21] Long, Y.C., Glund, S., Garcia-Roves, P.M., Zierath, J.R.: Calcineurin regulates skeletal muscle metabolism via coordinated changes in gene expression. J Biol Chem **282(3)**, 1607–14 (2007)

[22] Fleskens, V., Mokry, M., van der Leun, A.M., Huppelschoten, S., Pals, C.E., *et al.*: FOXP3 can modulate TAL1 transcriptional activity through interaction with LMO2. Oncogene **35(31)**, 4141–8 (2016)

[23] Wang, L., Jia, Y., Rogers, H., Wu, Y.P., Huang, S., Noguchi, C.T.: GATA-binding protein 4 (GATA-4) and T-cell acute leukemia 1 (TAL1) regulate myogenic differentiation and erythropoietin response via cross-talk with Sirtuin1 (Sirt1). J Biol Chem **287(36)**, 30157–69 (2012)

[24] Sanchez, A.M., Csibi, A., Raibon, A., Docquier, A., Lagirand-Cantaloube, J., *et al.*: eIF3f: a central regulator of the antagonism atrophy/hypertrophy in skeletal muscle. Int J Biochem Cell Biol **45(10)**, 2158–62 (2013)

[25] Bruscoli, S., Donato, V., Velardi, E., Di Sante, M., Migliorati, G., *et al.*: Glucocorticoid-induced leucine zipper (GILZ) and long GILZ inhibit myogenic differentiation and mediate anti-myogenic effects of glucocorticoids. J Biol Chem **285(14)**, 10385–96 (2010)

[26] L, G.-G., Vila-Bedmar, R., Carrasco-Rando, M., M., C.-S., M., M., *et al.*: Skeletal muscle myogenesis is regulated by G protein-coupled receptor kinase 2. J Mol Cell Biol **299-311**, 6–4 (2014)

[27] Holt, L.J., Turner, N., Mokbel, N., Trefely, S., Kanzleiter, T., *et al.*: Grb10 regulates the development of fiber number in skeletal muscle. FASEB **26(9)**, 3658–69 (2012)

[28] Ramazzotti, G., Billi, A.M., Manzoli, L., Mazzetti, C., Ruggeri, A., *et al.*: IPMK and b-catenin mediate PLC-b1-dependent signaling in myogenic differentiation. Oncotarget **7(51)**, 84118–84127 (2016)
